# A hidden Markov model for decoding and the analysis of replay in spike trains

**DOI:** 10.1007/s10827-016-0621-9

**Published:** 2016-09-13

**Authors:** Marc Box, Matt W. Jones, Nick Whiteley

**Affiliations:** 1 Bristol Centre for Complexity Sciences, University of Bristol, Bristol, UK; 2 School of Physiology and Pharmacology, University of Bristol, Bristol, UK; 3 School of Mathematics, University of Bristol, Bristol, UK

**Keywords:** Spike train modelling, Hidden markov model, Hippocampus, Decoding, Spike train replay

## Abstract

We present a hidden Markov model that describes variation in an animal’s position associated with varying levels of activity in action potential spike trains of individual place cell neurons. The model incorporates a coarse-graining of position, which we find to be a more parsimonious description of the system than other models. We use a sequential Monte Carlo algorithm for Bayesian inference of model parameters, including the state space dimension, and we explain how to estimate position from spike train observations (decoding). We obtain greater accuracy over other methods in the conditions of high temporal resolution and small neuronal sample size. We also present a novel, model-based approach to the study of replay: the expression of spike train activity related to behaviour during times of motionlessness or sleep, thought to be integral to the consolidation of long-term memories. We demonstrate how we can detect the time, information content and compression rate of replay events in simulated and real hippocampal data recorded from rats in two different environments, and verify the correlation between the times of detected replay events and of sharp wave/ripples in the local field potential.

## Introduction

### Background and motivation

This article is concerned with the development of statistical modelling techniques for multiple concurrent spike trains recorded from behaving rats using implanted microelectrodes. We are interested in data sets that include other variables, for example position in a maze, that may be correlated with concurrent spike trains. We focus on two applications relevant to this context: the *decoding* of position information encoded in hippocampal spike trains and the detection and analysis of spike train *replay*.

#### Decoding

Decoding is the task of estimating the information content transmitted by spike trains: sequences of times of spikes, or action potentials, recorded from individual neurons and considered as instantaneous and identical events (Rieke et al. 1999). Decoding has been used for the study of place cells: pyramidal cells of the hippocampus that spike selectively in response to the animal’s position O’Keefe and Dostrovsky (1971), O’Keefe (1976). Individual cells have been observed to encode collectively entire environments in this manner (“population coding” of space, Wilson and McNaughton (1993)). With large scale, parallel microelectrode recordings (Buzsáki 2004) it is possible to accurately decode the trajectory of an animal around an environment from population activity, with increasing accuracy as more cells are sampled (Zhang et al. 1998). In this article, *position* is the variable of interest for encoding and decoding, but these ideas can be applied more generally to other sensory or behavioural variables.

#### Replay

*Replay* is the reoccurrence of population spiking activity associated with a specific stimulus (an association made *online*: when the stimulus was presented), during times of unrelated behaviour (*offline*: times of sleep or motionlessness). The phenomenon has been most extensively studied in the place cells of rodents, in which spike trains encoding the trajectory of the animal are replayed in this manner. The time of hippocampal replay events has been found to correlate with the time of local field potential (LFP) events known as sharp wave/ripples (SWR, Buzsáki et al. (1992)), by Foster and Wilson (2006), Diba and Buzsáki (2007) and Davidson et al. (2009) during awake restful behaviour, and by Kudrimoti et al. (1999) during sleep.

Place cell replay has been demonstrated to occur on a faster timescale than the encoded trajectory: 20 times faster for cells of the hippocampus (Nádasdy et al. (1999),Lee and Wilson (2002)) and 5 to 10 times faster for cells of the cortex (Ji and Wilson (2006), Euston et al. (2007)). In the hippocampus this compression of spiking activity may be due to the burst firing of cells induced by SWR events (Csicsvari et al. (1999)), or the coordination of place cells by the LFP theta rhythm (O’Keefe and Recce (1993)), but it is not clear what is responsible for the effect in the cortex (Buhry et al. (2011)).

Although replay, and in particular *preplay* - the expression of offline behavioural sequences *prior* to the behaviour (Diba and Buzsáki (2007), Dragoi and Tonegawa (2011)) - have been suggested to play a role in active cognitive processes (Gupta et al. (2010), Pfeiffer and Foster (2013)), most of the literature concerned with the role of replay has focussed on the *consolidation hypothesis* (O’Neill et al. (2010), Carr et al. (2011)): that experiences are encoded online by cell assemblies in the hippocampus, then transmitted to the cortex for long-term storage during offline replay. This is supported by observations that hippocampal SWR coincide with high frequency oscillations in the cortex (Siapas and Wilson (1998), Mölle et al. (2006)), by observations of coordinated activation of cortical cells during hippocampal replay (Ji and Wilson (2006), Euston et al. (2007), Peyrache et al. (2009)), and by slowing of learning by blocking SWRs (Girardeau et al. (2009), Ego-Stengel and Wilson (2010)). Furthermore, correlated offline spiking patterns between pairs of cells within and between the hippocampus and cortex has been observed by Qin et al. (1360) and Sutherland and McNaughton (2000). However, it remains to be demonstrated whether the same encoded information is being replayed within the two regions during replay events, as implied by the consolidation hypothesis.

### Current approaches to decoding and replay detection

A simple statistical model used for decoding was described by Zhang et al. (1998) and compared favourably with nonparametric methods. This model, which we will refer to as the *Bayesian decoder (BD)*, has been influential in spike train analysis in general (Chen (2013)) and replay analysis in particular (e.g. in Davidson et al. (2009), Karlsson and Frank (2009), Dragoi and Tonegawa (2011), Pfeiffer and Foster (2013), and Wikenheiser and Redish (2013)). It consists of a parametric model for the number of spikes in consecutive time intervals, with position encoded as the expected spike count in each interval. Parameter values are estimated from a data set of observed spike trains and position using the method of maximum likelihood, and decoding is achieved by positing a prior distribution for position and using Bayes’ theorem to derive the posterior distribution over position given spike train observations. The BD approach to decoding is used as a performance benchmark in Section 3.2.

More complex models have attempted to account for the strong dependence through time of processes such as the trajectory of an animal and its concurrent spike trains in order to achieve greater accuracy of representation and decoding. In the state space model of Brown et al. (1998), and in the hidden Markov model (HMM) of Johnson and Redish (2007), spike counts are conditionally independent observations given the position, which constitutes a latent process. That is, a Markovian dependence structure is assumed for the position process, characterised by a transition matrix and initial state distribution. The spike train model is identical to that of BD. We refer to this model as the *latent position (LP)* hidden Markov model.

In the application of the HMM presented in Johnson and Redish (2007), the state space is determined by the set of positions explored, which may constitute far greater model complexity than is sufficient to characterise the spike train observations, thus incurring a greater computational burden and requiring more data in order to estimate the extra parameters. In Chen et al. (2012), an HMM is employed in which the state space is not identified with the set of positions (but is interpreted as a “virtual environment”). Parameters of the Markov chain are estimated from spike train observations only, rather than direct observations of the hidden process as in Johnson and Redish (2007). The number of states required to sufficiently characterise observations is determined through a process of model selection. Thus, Chen et al. (2012) are able to elicit directly from a spike train ensemble the distinct patterns of activity in place cells that may encode position, without needing to prespecify the receptive fields of these cells (the *place fields*, as would be necessary in a nonparametric approach), and to infer from the transition matrix the “topology” of the spatial representation. More recently, Linderman et al. (2016), have used Dirichlet process techniques to handle the unknown number of states in a hidden Markov model.

Replay has previously been detected as the improved correlation of cell pair firing rates post-behaviour by Pavlides and Winson (1989), Wilson and McNaughton (1994), and Skaggs and McNaughton (1996), and by using pattern-matching techniques in spike trains by Nádasdy et al. (1999) and Louie and Wilson (2001). More recently, statistical model-based decoding techniques such as BD have allowed researchers to begin to ask questions about replay directly in terms of the observable that is supposed to be encoded rather than purely as a spike train phenomenon: whether replay is preferentially of trajectories of a certain length, complexity or location, for example.

As well as specifying criteria for replay detection, other authors have found it necessary to guard against mistakenly detecting replay by chance (a type I error in the language of hypothesis testing). To this end, Davidson et al. (2009), Dragoi and Tonegawa (2011) and Pfeiffer and Foster (2013) used informal hypothesis testing to demonstrate positive discovery at a nominated statistical significance level. These tests are informal since the distribution of their test statistic under the null hypothesis (of no replay) is unknown, and hence it is not clear how to calculate a *p*-value. This is resolved in these studies by the use of a permutation test (or “Exact test”, Good (2005)), in which the unknown distribution is arrived at simply by evaluating the test statistic under all possible permutations of the test data. Since this is infeasible for candidate replay events of nontrivial length, a Monte Carlo version is typically used, in which a random sample of the test statistic is obtained via shuffling procedures on the test data. This approach comes with its own uncertainty: the “Monte Carlo *p*-value” is an approximate *p*-value when the sample taken is not exhaustive.

### The contributions of this article

#### **Model relating place cell spike trains to position**

We present a new statistical model, the *observed position (OP)* model, that offers improved performance for decoding and for the study of replay over the BD and LP models. Like Chen et al. (2012) we posit an HMM structure with an unobserved latent process to characterise the variation in observed processes. The difference between our model and that of Chen et al. (2012) is that we represent position as an observation process in parallel to the spike trains, allowing us to perform decoding when position data is missing, as in BD and LP.

Moreover, a particular innovative feature of our model is that it also uses latent state variables to model the evolution of position, effectively ‘coarse-graining’ physical locations and corresponding parameters of spiking activity. The first advantage of this approach is interpretational: in our model latent variables serve to associate clusters of statistically similar spiking activity with regions of the physical environment. The second advantage is that the number of state-variables does not necessarily scale up with spatial resolution of position measurements, but instead is treated as an unknown and inferred from the data. Thirdly, our model has a richer dependence structure than the BD and LP models, since upon marginalizing over the latent state variables in the OP model, the joint process of positions and spiking activity is non-Markovian, as too is the marginal process of positions. This compares to independence and Markovian assumptions in the BD and LP models, respectively. In numerical experiments we find the OP model can achieve better performance in decoding than the BD and LP models when we use a high time resolution and when we have spike trains from a small number of cells.

#### **A Bayesian inference algorithm for parameters and model size**

We make use of a sequential Monte Carlo (SMC) algorithm to perform Bayesian parameter inference, with a state space transformation suggested by Chopin (2007) to make the HMM identifiable. This algorithm makes a numerical approximation (achieving greater accuracy with larger SMC sample size) to the exact posterior distribution over parameters. This is in contrast to the the variational Bayes method used by Chen et al. (2012), which only targets approximations to the exact posterior distributions, induced by independence assumptions, and hence returns approximate parameter estimates. In addition to estimation of parameters, our algorithm also makes simultaneous inference for the number of latent states of the model.

#### **New methods for the analysis of replay**

We introduce a method for detecting replay of specific trajectories on different time scales, building directly on our model based decoding framework. We are able to compare the times of replay events for particular trajectories that may vary in spatial characteristics, duration and compression in time relative to behaviour. These properties of our methods make them useful in particular for exploring evidence that the information content of replay is coordinated between different neuronal populations, such as the hippocampus and neocortex.

In studies of replay such as Davidson et al. (2009), a model is used to decode a trajectory in the sense of computing a point estimate, which is then tested against criteria that constitute an operational definition of replay. Our advancement is to recognise in the model a description of all trajectories that *might* be encoded captured through the posterior distribution over positions given spike trains. We thus make full use of the information contained in the posterior distribution rather than only taking from it a point estimate. Moreover we do not need to resort to ad-hoc tests of statistical significance or the kind of shuffling procedures mentioned above nor do we need to accept any approximate *p*-values of uncertain accuracy.

### Structure of the article

Section 2 describes our data (Section 2.1) and our model (Sections 2.2 and 2.3), explains how we perform inference for model parameters, hidden states, and missing position data (decoding) (Section 2.4), and explains the analysis of replay within our model, including inference for the time and content of replay (Section 2.5). Also is explained how we detect SWR events and demonstrate correlation with replay events using the cross correlogram (Section 2.6) and the simulation of data (Section 2.7). Section 3 presents results from applying our model to simulated and real (experiment-generated) data. Model fitting results which demonstrate the model’s characterisation of spike train and position data are presented (Section 3.1), also the results of decoding position comparing our model against the BD and LP alternatives (Section 3.2), and our analysis of replay in simulated and real sleep data (Section 3.3). These results are discussed, and our methods appraised, in Section 4.

## Methods

### Description of the experimental data

Our experimental data sets consist of simultaneous recordings of a rat’s position and hippocampal spike trains. Two environments were used: a straight linear track and a double-ended T-maze (see Jones and Wilson (2005) for details). In each of these, a rat performed repeated consecutive trials of a reinforced learning task. In the linear track this consists in running from one end to the other, where food reward is received. In the T-maze the rat runs between rest sites in the terminal ends of corridors on opposite sides of the maze. Food reward is received at these sites, but on one side of the maze only when the correct corridor away from the “T” junction is chosen, reliably determined by recent experience.

In both experimental setups, two epochs of different behavioural conditions were used: a *RUN* epoch, in which the animal performed the learning task in the environment, immediately followed by a *REST* epoch, in which the animal remained in a separate dark box, in a state of quiescence likely including sleep. Spike trains were recorded from up to 19 hippocampal place cells throughout both epochs, and position in the environment was recorded using an infrared camera. Thus, for each environment we have a RUN data set (of spike trains and position) which we use for model parameter inference and for decoding analysis, and a REST data set (of spike trains only) which we use for replay analysis.

### Modelling

This section describes the OP model: a parametric model for discretised spike trains and position observations related via a hidden discrete time Markov chain. The model structure and parameterisation are explained in Sections 2.2.2 and 2.2.3. Section 2.2.4 addresses the identifiability of model parameters.

#### Data discretisation

Our spike train data consists of observations from *C* distinct point processes in continuous time. We use a time interval width *δt* seconds to partition this data into *T* time bins, and we let *Y*_*t*, *n*_ for 1 ≤ *n* ≤ *C* and 1 ≤ *t* ≤ *T* represent the number of times neuron *n* spikes in the *t* ^th^ time bin. We denote the random vector of spike counts from each neuron at time *t* as **Y**_*t*_, and we denote a time vector of variables between time bins *t*_1_ and *t*_2_ inclusive as $\mathbf {Y}_{t_{1}:t_{2}}$. We use the lowercase, as in $\mathbf {Y}_{t_{1}:t_{2}}$, to represent observed spike counts.

We use *X*_*t*_, for 1 ≤ *t* ≤ *T*, to denote the random discrete position of the animal in time bin *t*. Our position data consists of a sequence of two dimensional pixel coordinates recorded at a frequency of 25Hz. This will exceed any frequency implied by *δt* we use; therefore we can easily adapt these data to our discrete time scale of *T* time bins by taking the first observation in each bin.

We discretise space so that each *X*_*t*_ is a finite random variable. The raw two dimensional pixel coordinates are partitioned into a square grid; we then mark as inaccessible all grid squares covering regions outside of the maze. The remaining squares we label arbitrarily from 1 to *M*, forming the domain of *X*_*t*_.

#### HMM to relate spike trains to position

**Fig. 1:**
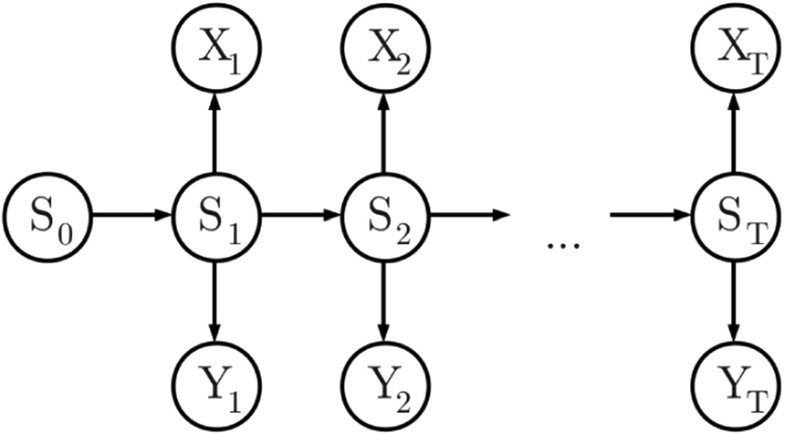
DAG for the LP model, explained in Section 2.2

We posit a discrete time Markov chain with *κ* states underlying the observation processes, denoted *S*_0:*T*_, with transition matrix $\mathbf {P} = \left (P_{i, j} \right )$ where $P_{i, j} :\!= Pr\left (S_{t} = j \mid S_{t - 1} = i \right )$ for 1 ≤ *i*, *j* ≤ *κ* and for all 1 ≤ *t* ≤ *T*, and initial state distribution $\mathbf {\pi } = \left (\pi _{i} \right )$ where $\pi _{i} :\!= Pr\left (S_{0} = i \right )$ for 1 ≤ *i* ≤ *κ*. The dependence between observation variables and the Markov chain is depicted in the directed acyclic graph (DAG) of Fig. 1.

We assume *Y*_*t*, *n*_ and *X*_*t*_ are conditionally independent of **Y**_1:*t*−1, *n*_, **Y**_*t*+1:*T*, *n*_, *X*_1:*t*−1_, *X*_*t*+1:*T*_, *S*_0:*t*−1_ and *S*_*t*+1:*T*_ for each *t*, given *S*_*t*_, so the joint probability of all model variables factorises as

1 $$ p \left(\mathbf{y}_{1:T}, x_{1:T}, s_{0:T} \mid \mathbf{\theta}, \kappa \right) = \pi_{s_{0}} \prod\limits_{t = 1}^{T} p \left(\mathbf{y}_{t}, x_{t} \mid s_{t}, \mathbf{\theta}, \kappa \right) P_{s_{t - 1}, s_{t}}, $$

in which **𝜃** represents the set of all model parameters. We further assume the conditional independence of *Y*_1:*T*, *n*_ for spike trains 1 ≤ *n* ≤ *C* and positions *X*_1:*T*_ given *S*_1:*T*_, so the likelihood factorises as

2 $$ p \left(\mathbf{y}_{t}, x_{t} \mid s_{t}, \mathbf{\theta}, \kappa \right) = p \left(x_{t} \mid s_{t}, \mathbf{\theta}, \kappa \right) \prod\limits_{n = 1}^{C} p \left(y_{t, n} \mid s_{t}, \mathbf{\theta}, \kappa \right). $$

We note that under these model assumptions, upon marginalizing over the state-variables in (1), the bi-variate process of positions and spike-counts, (*X*_1_, **Y**_1_),(*X*_2_, **Y**_2_),… is non-Markovian.

#### Parametric observation models

##### **Spike trains**

We model our discrete spike trains *Y*_1:*T*, *n*_ as Poisson random variables with piecewise constant means and with jumps between means on changes of state of the Markov chain. That is, we posit *κ* distinct Poisson rates for each spike train, denoted λ_*i*, *n*_ for 1 ≤ *i* ≤ *κ* and 1 ≤ *n* ≤ *C*. Thus $Y_{t, n} \mid S_{t} = s \sim \mathtt {Poi}\left (\delta t \lambda _{s, n} \right )$, and

3 $$ p \left(Y_{t, n} = y_{t, n} \mid S_{t} = i, \mathbf{\theta}, \kappa \right) = e^{-\delta t \lambda_{i, n}} \frac{ \left(\delta t \lambda_{i, n} \right)^{y_{t, n}} }{ y_{t, n}! }. $$

##### **Position**

We model *X*_*t*_ using *κ* distinct categorical distributions, labelled by *S*_*t*_, over the set of outcomes $\{1, 2, \dots , M\}$ that jump in parallel with the spike train processes. Outcomes of the *i* ^th^ distribution are explained by an underlying two dimensional Gaussian with mean *ξ*_*i*_ and covariance matrix Σ_*i*_. These are the only free parameters of the position model.

This is achieved by mapping discrete positions 1 to *M* to the Euclidean plane using a transformation that preserves the topology of the maze, as follows. We define a distance function $\bar {d}: \{ 1, 2, \dots , M \} \times \{ 1, 2, \dots , M \} \to \mathbb {R}$ that returns the distance between two positions when access from one to the other is constrained to traversable maze regions (i.e. along corridors). This is achieved by measuring the distance cumulatively through adjacent positions. We use the transformation $\mathbf {f}_{x}: \{1, 2, \dots , M\} \to \mathbb {R}^{2}$ to map discrete positions *x* ^′^ to vectors in $\mathbb {R}^{2}$ of length $\bar {d}(x, x^{\prime })$; details are given in Appendix A. The categorical probabilities for our discrete position model are then

4 $$ p \left(X_{t} = x \mid S_{t} = i, \xi_{i}, \Sigma_{i} \right) = \frac{q \left(\mathbf{f}_{\xi_{i}} \left(x \right); 0, \Sigma_{i} \right)}{{\sum}_{x^{\prime} = 1}^{M} q\left(\mathbf{f}_{\xi_{i}} \left(x^{\prime} \right); 0, \Sigma_{i} \right)}, $$

where

5 $$ q \left(\mathbf{f}_{\xi_{i}} \left(x \right); 0, \Sigma_{i} \right) = \exp \left\{ \mathbf{f}_{\xi_{i}} \left(x \right)^{\intercal} \Sigma^{-1}_{i} \mathbf{f}_{\xi_{i}} \left(x \right)\right\}, $$

the unnormalised probability density of the two dimensional Gaussian distribution with mean 0 and covariance matrix Σ_*i*_ evaluated at $\mathbf {f}_{\xi _{i}} \left (x \right )$.

The purpose of this general approach is that we obtain a position model that satisfies our intuition for the accessibility of places from each other in non-convex environments such as a T-maze. In particular the distribution over *X*_*t*_ given a particular state should be unimodal, having monotonically decreasing probability with distance from the modal position, since positions of similar probability should be local. This is violated in a concave environment when using the Euclidean distance in place of *d*.

By thus constraining the categorical outcome probabilities, we reduce the number of free parameters from *M*−1 for each state to simply a modal position *ξ*_*i*_ and a covariance matrix Σ_*i*_ for each state. Therefore, unlike in the LP model, in OP we are free to choose any spatial resolution *M* (up to the resolution of raw observations) without causing undersampling problems or high computational cost due to the effect on the state space. No free parameters are introduced by increasing the spatial resolution.

#### Augmented Markov chain for model identifiability

The model described above is not identifiable because there are subsets of parameters that are exchangeable in prior distribution and which under arbitrary permutations of the state label leave the likelihood invariant (Scott 2002). This is the case for $\{\lambda _{1, n}, \lambda _{2, n}, \dots , \lambda _{\kappa , n}\}$ for each *n* and for $\{\xi _{1}, \xi _{2}, \dots ,\linebreak \xi _{\kappa }\}$. We make use of a reformulation of the model suggested by Chopin (2007) to make the model identifiable, and which also readily accommodates inference for *κ*.

Since state labels are arbitrary, we can relabel states in order of their appearance in the Markov chain *S*_0:*T*_ without affecting the model structure. This ordering of states in relation to the data means that permutations of exchangeable parameters will not leave the likelihood invariant. The relabelling is realised via the parameterisation of the Markov chain with an extension to its state space. For sequential relabelling, *s*_0_ = 1, so we must have *π*_1_ = 1 and *π*_*i*_ = 0 for 2 ≤ *i* ≤ *κ*. We must then keep track of the number of distinct states emitted up to any time step *t*. That is, if we have *S*_*t*_ = *i* ≤ *K*<*κ*, we must impose the restriction that *S*_*t*+1_ ≤ *K*+1, with equality if and only if *S*_*t*+1_ has not been emitted before time *t*+1. Thus, we let random variable *K*_*t*_, taking values in $\{1, 2, \dots , \kappa \}$, be the number of distinct states emitted up to and including time *t*.

We can now define the augmented process $\widetilde {S}_{0:T}$ constituted by the sequence of random variables $\widetilde {S}_{t} \equiv \left (S_{t}, K_{t} \right )$, which have $\widetilde {\kappa } = \frac {\kappa \left (\kappa + 1 \right )}{2}$ distinct outcomes (since values are constrained by *S*_*t*_ ≤ *K*_*t*_ ≤ *κ*). This process is a Markov chain with transition matrix $\mathbf {\widetilde {P}} = \left (\widetilde {P}_{i, j} \right )$ for $1 \le i, j \le \widetilde {\kappa }$. If we let $i \equiv \left (s^{\prime }, k^{\prime } \right )$, $j \equiv \left (s^{\prime \prime }, k^{\prime \prime } \right )$, with $s^{\prime }, s^{\prime \prime }, k^{\prime }, k^{\prime \prime } \in \{1, 2, \dots , \kappa \}$, we have

6 $$ \widetilde{P}_{i, j} = \left\{\begin{array}{lll} P_{s^{\prime}, s^{\prime\prime}} & \text{if } s^{\prime}, s^{\prime\prime} \le k^{\prime\prime} = k^{\prime} \le \kappa,\\ \sum\limits_{s = k^{\prime} + 1}^{\kappa} P_{s^{\prime}, s} & \text{if } s^{\prime\prime} = k^{\prime\prime} = k^{\prime} + 1 \le \kappa,\\ 0 & \text{else}. \end{array}\right. $$

The first case of Eq. (6) corresponds to a transition between two states previously emitted. The second to emitting a new state: since states are mutually exclusive outcomes of *S*_*t*_ the probability of transitioning from some state $s^{\prime }$ to any of the previously unseen states is the sum of the transition probabilities from $s^{\prime }$ to each unseen state. The last case covers the violations of the above constraints.

Observations *X*_*t*_ and **Y**_*t*_ are considered conditionally independent of *K*_*t*_ given *S*_*t*_ for 1 ≤ *t* ≤ *T*, so this reparameterisation does not alter the dependence structure between state and observation variables of Fig. 1.

### Priors and full conditionals

This section describes prior distributions and full conditional distributions for the model parameters. These are required for the posterior sampling of parameters as part of the SMC algorithm for Bayesian parameter inference and model selection, explained in Appendix C.

We assume a hierarchical model structure with the following factorisation for the prior of **𝜃** and *κ*:

7 $$ p \left(\mathbf{\theta}, \kappa \mid \mathbf{\phi} \right) = p \left(\mathbf{\theta} \mid \kappa, \mathbf{\phi} \right) p \left(\kappa \mid \mathbf{\phi} \right), $$

in which **ϕ** is the set of all hyperparameters. This allows us to efficiently sample $\left (\mathbf {\theta }, \kappa \right )$ by first sampling *κ*. This task is facilitated by assuming that model parameters in **𝜃**, with **P** considered as *κ* row vectors **P**_*i*,⋅_, are conditionally independent of each other given *κ* and **ϕ**. This gives us the factorisation

8 $$\begin{array}{@{}rcl@{}} p \left(\mathbf{\theta} \mid \kappa, \mathbf{\phi} \right) &= &p \left(\mathbf{\pi} \mid \kappa, \mathbf{\phi} \right) \\ && \times \prod\limits_{i = 1}^{\kappa} p \left(\mathbf{P}_{i, \cdot} \! \mid\! \kappa, \mathbf{\phi} \right) p \left(\xi_{i} \!\mid\! \kappa, \mathbf{\phi} \right) p \left(\Sigma_{i} \mid \kappa, \mathbf{\phi} \right) \\ &&\times \prod\limits_{n = 1}^{C} p \left(\lambda_{i, n} \mid \kappa, \mathbf{\phi} \right), \end{array} $$

and thus we may sample each parameter from its respective marginal prior independently, conditional on a value for *κ*. For each marginal prior we use a distribution conjugate to the relevant likelihood function, to facilitate sampling using standard distributions, and we fix all hyperparameters with constant values that give rise to uninformative priors.

For *κ*, we assume a discrete uniform prior with parameter $\bar {\kappa } \in \mathbf {\phi }$, a positive integer. That is, *κ* can take on values a priori at random between 1 and $\bar {\kappa }$. This expresses a lack of prior information regarding the model complexity, up to an upper limit. We must choose $\bar {\kappa }$ to be great enough that all model sizes that may be appropriate to the data are possible, but we are subject to increasing computational costs with larger $\bar {\kappa }$. Appropriate values can be arrived at by initial exploratory runs of the algorithm in Appendix C; besides this upper limit, we find in practise that the form of the prior (uniform) has little effect on the posterior.

Priors for each parameter in *𝜃* are described in the remainder of this section along with a discussion of the corresponding full conditionals, $p \left (\vartheta \mid x_{1:t}, \mathbf {y}_{1:t}, s_{0:t}, \mathbf {\theta } \setminus \vartheta , \kappa , \mathbf {\phi } \right )$ for some variable *𝜗*∈*𝜃*, restricted to time *t*. Note we are not required to sample parameters of the initial state distribution **π** because the initial state is fixed at 1 (cf. Section 2.2.4).

#### **Firing rates**

For the mean firing rates λ_*i*, *n*_ we take a Gamma prior Gam(λ_*i*, *n*_;*α*, *β*), with shape parameter *α* and rate parameter *β*, which is the conjugate prior for these parameters. Values of $\alpha = \frac {1}{2}, \beta = 0$ correspond to the uninformative Jeffreys prior (Gelman et al. (2003), p69). This prior is improper and cannot be sampled from, so we use *β* = 0.01 for a practical alternative that is largely uninformative.

ution for λ_*i*, *n*_ at time step *t* is Gam(λ_*i*, *n*_;*α* ^∗^, *β* ^∗^) with

9 $$ \alpha^{*} = \sum\limits_{u \le t: s_{u} = i} y_{t, n} + \alpha, $$

10 $$ \beta^{*}= \delta t c_{i, t} + \beta,$$

where $c_{i, t} :\!= {\sum }_{u = 1}^{t} \bullet \{s_{u} = i\}$; see Appendix B.1 for derivation.

#### **Position model modes**

For the position hyperparameter *ξ*_*i*_ we use as prior the discrete uniform distribution over positions 1 to *M*. Note that we could consider *ξ*_*i*_ as the mean of a Gaussian distribution, for which a Gaussian distribution is the conjugate prior, but for sampling from an uninformative prior with our discretisation of positions the uniform distribution is equivalent and simpler.

The full conditional distribution has the same form as the likelihood, since

11 $$\begin{array}{@{}rcl@{}} p \left(\xi_{i} \mid x_{1:t}, \mathbf{y}_{1:t}, s_{0:t}, \mathbf{\theta}, \kappa, \mathbf{\phi} \right) &\propto & p \left(x_{1:t} \!\mid s_{0:t}, \mathbf{\theta}, \kappa, \right) p \left(\xi_{i} \mid \mathbf{\phi}, \kappa \right) \\ &\propto & p \left(x_{1:t} \mid s_{0:t}, \mathbf{\theta}, \kappa, \right) \\ &\propto & \prod\limits_{u \le t: s_{u} = i} p \left(x_{u} \mid i, \xi_{i}, \Sigma_{i} \right), \end{array} $$

and furthermore

12 $$ p \left(x_{u} \mid i, \xi_{i}, \Sigma_{i} \right) \propto q \left(\mathbf{f}_{\xi_{i}} \left(x_{u} \right); 0, \Sigma_{i} \right) $$

by Eq. (4), so the posterior is $\texttt {N} \left (\mathbf {f}_{\xi ^{*}} \left (\xi _{i} \right ); 0, \Sigma ^{*} \right )$ with

13 $$\begin{array}{@{}rcl@{}} \xi^{*} &= & \bar{x}_{i} \in \arg\min\limits_{x \in \{1, 2, \dots, M\} } \left\{c_{i, t}^{-1} {\sum}_{u \le t: s_{u} = i} \mathbf{f}_{x} \left(x_{u} \right)\right\}, \end{array} $$

14 $$\begin{array}{@{}rcl@{}} \Sigma^{*} &= & c_{i, t}^{-1} \Sigma_{i}, \end{array} $$

which is derived in Appendix B.2. Via this construction we can sample *ξ*_*i*_ from the categorical distribution with probabilities obtained from $\texttt {N} \left (\mathbf {f}_{\xi ^{*}} \left (\xi _{i} \right ); 0, \Sigma ^{*} \right )$ and normalised as in Eq. (4).

#### **Position model covariance matrices**

We use the conjugate Inverse-Wishart distribution as prior for Σ_*i*_, with parameters Ψ and *δ*. This prior expresses our conception of how states characterise variability in size and shape of the regions represented in our model. These regions can be likened to place fields but for a population of place cells: they emerge from the collective activity of multiple cells. This interpretation may guide our parameterisation of this prior, since it is difficult to specify an uninformative prior over covariance matrices. The hyperparameter Ψ is the 2×2 positive definite matrix of sums of squared deviations of positions transformed by $\mathbf {f}_{\xi _{i}}$, a priori, and *δ* is the degrees of freedom of the data from which Ψ was derived. Thus, Ψ can be set to encode our indifference to orientation or skewness of regions represented by each state by putting Ψ_1,1_ = Ψ_2,2_ and Ψ_1,2_ = Ψ_2,1_ = 0. This leaves Ψ_1,1_ free, to be set according to our prior conception of how large these regions typically are. The influence of this hyperparameter on the prior is weighted by *δ*; therefore a relatively uninformative prior is achieved by setting *δ* small (relative to the number of time bins in the data set). The full conditional for Σ_*i*_, also Inverse-Wishart by the conjugate relationship to the Gaussian likelihood with known mean, has parameters (Gelman et al. (2003), p87)

15 $$\begin{array}{@{}rcl@{}} \Psi^{*} & = & \Psi + SS_{i, t} \left(\xi_{i} \right) \end{array} $$

16 $$\begin{array}{@{}rcl@{}} \delta^{*} & = & \delta + c_{i, t}, \end{array} $$

where $SS_{i, t} \left (\xi _{i} \right )$ is the 2×2 matrix of sums of squared deviations around *ξ*_*i*_ in the transformed space,

17 $$ SS_{i, t} \left(\xi_{i} \right) :\!= \sum\limits_{u \le t: s_{u} = i} \mathbf{f}_{\xi_{i}} \left(x_{u} \right)^{\intercal} \mathbf{f}_{\xi_{i}} \left(x_{u} \right). $$

Note that in the full conditionals for *ξ*_*i*_ or Σ_*i*_, the other parameter is considered known. In sampling procedures, we therefore either sample *ξ*_*i*_ first conditional upon the value of Σ_*i*_ previously sampled, or vice versa.

#### **Rows of the transition matrix**

We use the Dirichlet prior for rows of **P**; that is, Dir(**P**_*i*,⋅_;**ω**). For an uninformative prior, we use a vector of *κ* ones for **ω**.

The structure we imposed on **P** (cf. Section 2.2.4) means the full conditional for a row **P**_*i*,⋅_ is a *Generalised Dirichlet distribution* rather than a standard Dirichlet distribution. At time step *t* this is derived as

18 $$\begin{array}{@{}rcl@{}} && p \left(\mathbf{P}_{i, \cdot} \mid x_{1:t}, \mathbf{y}_{1:t}, \widetilde{s}_{0:t}, \mathbf{\theta}, \kappa, \mathbf{\phi} \right) \\ &&\quad\propto p \left(s_{1:t} \mid k_{1:t}, \mathbf{\omega} \right) p \left(\mathbf{P}_{i, \cdot} \mid \mathbf{\omega}, \kappa \right) \\ &&\quad\propto {\underset{\underset{k_{u} = k_{u - 1}}{u \le t: s_{u - 1} = i, }}{\prod}} p \left(S_{u} = s_{u} \mid S_{u - 1} = i, \mathbf{P}_{i, \cdot} \right) \\ &&\qquad \times {\underset{ \underset{k_{u} = k_{u - 1} + 1}{u \le t: s_{u - 1} = i,}}{\prod}} p \left(S_{u} = s_{u} \mid S_{u - 1} = i, \mathbf{P}_{i, \cdot} \right) \\ &&\qquad \times p \left(\mathbf{P}_{i, \cdot} \mid \mathbf{\omega}, \kappa \right). \end{array} $$

Note we can ignore *s*_0_ because **π** is constant. The factorisation of $p \left (s_{1:t} \mid k_{1:t}, \mathbf {\omega } \right )$ in Eq. (18) follows from the Markov property; the first factor consists of transition probabilities between states previously emitted by the Markov chain, the second consists of transition probabilities to new states. Recall from Eq. (6) that these are treated differently. Continuing Eq. (18) we have

19 $$\begin{array}{@{}rcl@{}} & p& \left(\mathbf{P}_{i, \cdot} \mid x_{1:t}, \mathbf{y}_{1:t}, \widetilde{s}_{0:t}, \mathbf{\theta}, \kappa, \mathbf{\phi} \right) \\ &&\propto \prod\limits_{j = 1}^{\kappa} P_{i, j}^{ A_{i, j}(t) - B_{i, j}(t) } \prod\limits_{j = 1}^{\kappa} \left(\sum\limits_{l = j + 1}^{\kappa} P_{i, l} \right) ^ {B_{i, j}(t)} p \left(\mathbf{P}_{i, \cdot} \mid \mathbf{\omega}, \kappa \right) \\ &&=\prod\limits_{j = 1}^{\kappa} P_{i, j}^{ A_{i, j}(t) - B_{i, j}(t) + \omega_{j} - 1 } \left(\sum\limits_{l = j + 1}^{\kappa} P_{i, l} \right) ^ {B_{i, j}(t)}, \end{array} $$

where **A**(*t*) is the matrix of transition counts at time step *t*,

20 $$ A_{i, j}(t) := {\sum}_{u = 1}^t \mathbb{1} \{s_u = j, s_{u - 1} = i\} $$

and **B**(*t*) is the matrix of first arrival indicator variables at time step *t*,

21 $$ B_{i, j}(t) :\!= \left\{\begin{array}{ll} 1, \quad \text{the first \textit{j} in \(s_{1:t}\) immediately follows \textit{i},}\\ 0, \quad \text{else,} \end{array}\right. $$

for 1 ≤ *i*, *j* ≤ *κ*. The posterior probabilities given by Eq. (19) correspond to a Generalised Dirichlet distribution with parameters **ζ**_*i*_ = **A**_*i*,⋅_(*t*)−**B**_*i*,⋅_(*t*)+**ω** and **γ**_*i*_ = **B**_*i*,⋅_(*t*) (Wong 1998). We can use the algorithm of Wong (1998) to efficiently sample from this posterior; details are provided in Appendix B.3.

### Inference with our model

There are four kinds of inference we are interested in and can perform with our model. The first is inference for model parameters *𝜃*. Details of the Sequential Monte Carlo algorithm we use to estimate posterior distributions are given in Appendix C. Section 2.4.1 explains how we use the posterior expectation as point estimate for *𝜃*. Secondly, for states *S*_0:*T*_: this is explained in Section 2.4.2, in which is also also explained how we arrive at an estimate for *κ*. Thirdly, for position variables *X*_1:*T*_ from spike train observations **Y**_1:*T*_: *decoding* position, explained in Section 2.4.3. The fourth kind of inference is for the occurrence of replay in REST data. The analysis of replay is treated in Section 2.5.

#### Parameter estimation

The algorithm of Appendix C results in a sample approximation to $p \left (\mathbf {\theta }, \kappa \mid x_{1:T}, \mathbf {y}_{1:T}, \mathbf {\phi } \right )$, consisting of *H* samples $\{\theta ^{h},\kappa ^{h}\}_{h=1}^{H}$ and associated weights $\{w_{h}\}_{h=1}^{H}$. We make a point estimate $\hat {\mathbf {\theta }}$ of **𝜃** using the sample posterior mean. For a particular parameter *𝜗*_*i*_ associated with state *i*, we have

22 $$ \hat{\vartheta}_{i} = \frac{ {\sum}_{h: \kappa^{h} \ge i} w_{h} {\vartheta_{i}^{h}} }{ {\sum}_{h: \kappa^{h} \ge i} w_{h} }. $$

This achieves a marginalisation of *κ*.

#### State estimation

We use the smoothed posterior distributions over *S*_*t*_, *K*_*t*_ to estimate the state variable at each time step and the number of states *κ*. Inference for *κ* could be performed via Eq. (48) with an estimate $\hat {\kappa }$ taken as the mode; however, as argued in Chopin (2007) this is an estimate of how many states would be observed eventually if we took enough observations and one should use the posterior distribution of *K*_*T*_ to estimate how many distinct states were emitted during the *T* time steps. Thus, after fixing *𝜃* to our estimates $\hat {\theta }$, we use the forward-backward algorithm to compute the smoothed posterior distributions

23 $$ p \left(S_{t} = i, K_{t} = k \mid x_{1:T}, \mathbf{y}_{1:T}, \hat{\theta} \right), $$

for all $\left (i, k \right ) \in \left \{1, 2, \dots , \kappa \right \}^ 2$ and for all $t \in \left \{1, 2, \dots , T \right \}$. We obtain the marginal distribution over *K*_*t*_ by summing Eq. (23) over all $\bar {\kappa }$ values of *S*_*t*_, and vice versa for *S*_*t*_. The maximum a posteriori (MAP) estimate at time step *t* is the value that maximises the marginal posterior distribution. We take the MAP estimate of *K*_*T*_ for $\hat {\kappa }$, our estimate of the number of states required to characterise the data. We can alternatively use the Viterbi algorithm, described in Scott (2002), which returns the sequence $\widetilde {S}_{0:T}$ of greatest posterior probability, i.e. the sequence that maximises $p \left (\widetilde {s}_{0:T} \mid x_{1:T}, \mathbf {y}_{1:T}, \hat {\theta } \right )$.

Other methods to model size estimation have been used in a similar context, e.g. using the deviance information criterion (DIC) to compare models, or the DIC within a nonparametric extension of the HMM framework (Chen et al. 2012, 2014). We have no need for this, since the augmented states used to make the model identifiable already permit model size inference in the manner explained above.

#### Position decoding

We can also use our model to estimate (decode) position at any time from spike train observations. We can compute the position posterior distributions, $p \left (x_{t} \mid \mathbf {y}_{1:T}, \hat {\theta } \right )$, and hence obtain the MAP point estimate, as used by other authors in studies of replay such as Davidson et al. (2009). To do this we take advantage of the conditional independence of *X*_*t*_ from **Y**_1:*T*_ given *S*_*t*_, which permits

24 $$\begin{array}{@{}rcl@{}} p \left({\vphantom{\mathbf{y}_{1:T}, \hat{\theta}}} X_{t} \right.&=& \left.x, S_{t} = i \mid \mathbf{y}_{1:T}, \hat{\theta} \right) \\ &=& p \left(S_{t} = i \mid \mathbf{y}_{1:T}, \hat{\theta} \right) p \left(X_{t} \!= x \mid S_{t} = i, \hat{\xi}_{i}, \hat{\Sigma}_{i} \right).\\ \end{array} $$

On the right hand side of Eq. (24) is the marginal smoothing posterior at time step *t* using spike train observations only, and the conditional probability over positions given state, using the fitted model parameters. We then obtain the position posterior distribution by marginalising *S*_*t*_.

We can instead compute the trajectory $\hat {x}_{1:T}$ of greatest posterior probability, i.e. that maximises $p \left (x_{1:T} \mid \mathbf {y}_{1:T}, \hat {\theta } \right )$. For this we use a modified version of the Viterbi algorithm, explained in Appendix D.

### Model-based replay detection

In an analysis of sleep replay we wish to make three kinds of inference: the time of replay occurring, the information content being replayed, and the rate of time compression relative to the behavioural timescale. The methods described in this section allow us to achieve each of these.

Our idea is to use the posterior distribution over trajectories given spike train observations as a representation of what information is encoded at different times. We identify replay as occurring at a particular time when the posterior probability of a certain trajectory obtains a maximum above some threshold (see Section 2.5.1). For inference regarding the information content being replayed, we fix the trajectories to be used for this posterior evaluation. We call these *template* trajectories (Section 2.5.2). For the rate of temporal compression, we search for replay in temporally compressed data at many different compression rates (Section 2.5.3).

Spike train data for replay analysis may be distinct from the training data (for example when using a REST epoch for replay analysis) and therefore constitute dynamics and correlations that may not be described accurately by the model with $\mathbf {\theta } = \hat {\mathbf {\theta }}$ estimated from RUN. We must therefore demonstrate predictive power for our model with parameterisation $\hat {\mathbf {\theta }}$ on the data $\mathbf {y}^{\text {REST}}_{1:T}$, for which we use a likelihood-based method, explained in Section 2.5.4.

#### Replay score

We define the *replay score*, Ω, for template trajectory *x*_1:*a*_ at time *t*, as the ratio of likelihoods

25 $$ \Omega \left(x_{1:a}, t; \mathbf{y}_{1:T}, \mathbf{\theta} \right) :\!= \frac{ p \left(X_{t} = x_{1}, \dots, X_{t + a - 1} = x_{a} \mid \mathbf{y}_{1:T}, \mathbf{\theta} \right) }{ p \left(X_{t} = x_{1}, \dots, X_{t + a - 1} = x_{a} \mid \mathbf{\theta} \right) }. $$

An algorithm for computing the numerator of Eq. (25) is described in Appendix E, and for the denominator in Appendix F. Then we say that template *x*_1:*a*_ is *replayed at time* *t* ^*rep*^, on the discrete timescale, if

26 $$ \Omega = \Omega \left(x_{1:a}, t^{rep}; \mathbf{y}^{\text{REST}}_{1:T}, \hat{\mathbf{\theta}} \right) > \Omega^{*} $$

and

27 $$\begin{array}{@{}rcl@{}} \Omega > \max &\left\{\Omega \left(x_{1:a}, t^{rep} - 1; \mathbf{y}^{\text{REST}}_{1:T}, \hat{\mathbf{\theta}} \right) ,\right.\\ &\left. \qquad \Omega \left(x_{1:a}, t^{rep} + 1; \mathbf{y}^{\text{REST}}_{1:T}, \hat{\mathbf{\theta}} \right)\right\}, \end{array} $$

for some threshold Ω^∗^, where $\hat {\mathbf {\theta }}$ are the model parameters estimated from RUN. Since Eq. (25) has the form of a model likelihood ratio between the model for trajectories conditional on spike train observations and the model for trajectories marginal of spike trains, in our applications we use for Ω^∗^ values suggested by Kass and Raftery (1995) for likelihood ratios in Bayesian model comparison. Those authors provide useful interpretations for this ratio, in particular that Ω^∗^ = 20 is the minimum for “strong” evidence and Ω^∗^ = 150 for “very strong” evidence.

#### Templates

**Fig. 2:**
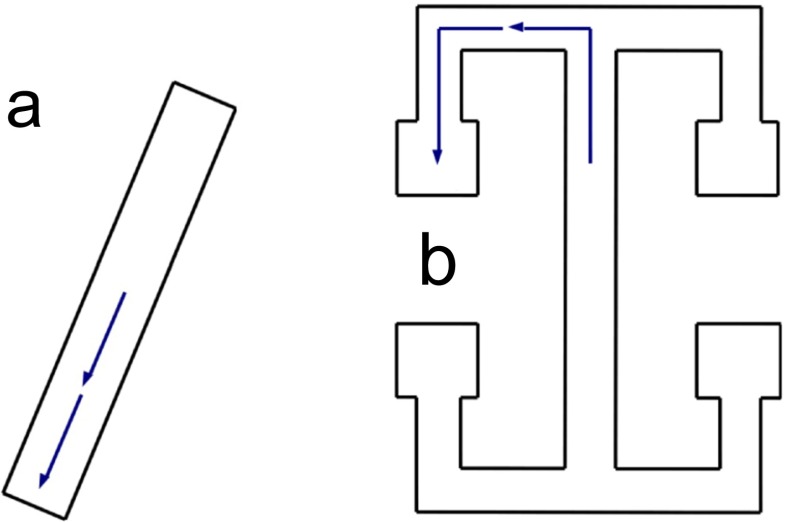
Top-down outline of the two environments used in RUN data (not to scale). Blue arrows represent example template trajectories used for replay detection. **a** linear track. **b** T-maze

We describe a collection of trajectories of the form *x*_1:*a*_ to use in Eq. (25). For the results presented in Section 3.3 we use segments of the RUN trajectory through particular regions of the environment; for example around a corner or into a rest site (on the T-maze). We chose segments running in both directions, i.e. towards and away from the centre of the environment. Examples of how these template trajectories might look are given in Fig. 2.

#### Time compression

By choosing templates that represent trajectories at uncompressed (behavioural) speeds, we are able to use our replay detection method for studying replay on a rapid (compressed) time scale relative to the behavioural time scale by adjusting the time discretisation bin width used for the analysis data. That is, for the detection of replay of a template *x*_1:*a*_ at compression rate *c*, we compute Ω using Eq. (25) on compressed spike train data $\overline {\mathbf {y}}_{1:cT}$ obtained by re-binning the raw spike train data, using the procedure of Section 2.2.1, with bin width $\overline {\delta t} = \delta t / c$.

#### Assessing model fit on analysis data

In order to justify our use of $\hat {\mathbf {\theta }}$ in Eq. (25), i.e. our model fitted to a RUN data set being used for replay detection on a REST data set, we make an assessment of model fit using the data likelihood (Gelman et al. 2003), $p \left (\mathbf {y}^{\text {REST}}_{1:T} \mid \mathbf {\theta }, \kappa \right )$. In particular we use the Bayesian information criterion (BIC, Schwarz 1978)

28 $$ BIC = -2 \log p \left(\mathbf{y}^{\text{REST}}_{1:T} \mid \mathbf{\theta}, \kappa \right) + N \log T, $$

where *N* is the number of free parameters in the model ($N = \hat {\kappa } \left (\hat {\kappa } + C + 3 \right )$ for OP). A lower BIC implies a better fit to the data, and includes a penalty for larger models. We compute the BIC for various parameterisations of the model: our estimates obtained from training (RUN) data, $\hat {\theta }$, and several alternatives chosen as benchmarks for particular aspects of model fit. Firstly, the model fitted to the analysis data itself, i.e. *𝜃* is estimated from REST spike train data using the procedure of Appendix C, ignoring the position model. We expect the BIC for $\hat {\theta }$ estimated from RUN to be greater than this alternative, but if it is close relative to an inferior benchmark we will have evidence that $\hat {\theta }$ estimated from RUN is well fit to REST. Secondly, as an inferior benchmark, we compute the BIC for a sample of *𝜃* drawn from the prior (cf. Section 2.3) and the BIC evaluated with *𝜃* set to the prior mean. Thirdly, the model with parameterisation $\hat {\theta }$ except for the transition matrix **P**; instead we assume that the states *S*_*t*_ are independent and each distributed according to the stationary distribution associated with **P**. This we use to assess whether the temporal dependence associated with parameters inferred from the training data is beneficial to the description of the analysis data. If this alternative has a lower BIC, it suggests the dynamics described by **P**, as estimated from the RUN data, do not also describe the REST data as well as simply assuming independence through time. Fourthly, BD, as described by Zhang et al. (1998) and with parameters estimated from RUN using maximum likelihood.

#### Replay detection algorithm

We can now state our replay detection algorithm as follows:

1. Use training data (a RUN epoch) and the procedure of Appendix C and 2.4.1 to estimate model parameters as $\hat {\mathbf {\theta }}$.
2. Use the model comparison approach of Section 2.5.4 to verify the fitted model can be used on the analysis (REST) data.
3. Construct a set of templates $\left \{x_{1:a_{r}}^{(r)}\right \}_{r = 1}^{R}$.
4. Evaluate Eq. (25) for each template $x_{1:a_{r}}^{(r)}$ and for $t = 1, \dots , T - a_{r} + 1$.
5. Report *t* ^*rep*^ as a replay of template *r* whenever Eqs. (26) and (27) are satisfied at *t* ^*rep*^ for $x_{1:a_{r}}^{(r)}$.

Times of replay events detected using this procedure at different compression rates are then classified as distinct events only when the extent of their temporal overlap is less than 50 *%*. This is necessary because the time of the event, as indicated by a local optimum of Ω, is liable to change between compression rates since slight adjustments to the placement of the template may improve the score. This rule is applied also to events detected using different templates: when two or more detected events overlapped by at least 50 *%*, the event with greatest Ω was retained and all others discarded, to prevent multiple discoveries of the same event.

### Correlation of replay with SWR events

We use the cross-correlogram between replay events and SWR events to demonstrate correlation between these two processes. SWR events were detected by bandpass filtering LFP between 120Hz and 250Hz, then taking the times of peak filtered LFP during intervals exceeding 3.5 standard deviations. In addition, we required that these intervals were between 30ms and 500ms in duration, between 20*μ*V and 800*μ*V in amplitude and with a gap between distinct intervals of at least 50ms.

The correlation between the process consisting of replay events (*rep*) and the process of SWR events (*rip*) at a temporal offset *u* seconds from any time *t* is measured by the second-order product density function for stationary point processes (Brillinger 1976),

29 $$\begin{array}{@{}rcl@{}} \rho_{rep, rip} \left(u \right) :\!= \lim\limits_{h, h^{\prime} \to 0} Pr &\left(rep \text{ event in } (t + u, t + u + h], \right.\\ &\qquad \left. rip \text{ event in } (t, t + h^{\prime}] \right) / h h^{\prime}.\\ \end{array} $$

An unbiased estimator of this is

30 $$ \hat{\rho}_{rep, rip} \left(u \right) = \left(\tau T \delta t \right)^ {-1} J_{rep, rip} \left(u \right) $$

(Brillinger 1976), in which $J_{rep, rip} \left (u \right )$ is the cross correlogram at lag *u* with bin width *τ*,

31 $$\begin{array}{@{}rcl@{}} &&J_{rep, rip} \left(u \right) :\!= \text{card} \left\{ \left(i, j \right) : u - \tau / 2 < t^{rep}_{i} - t^{rip}_{j} \!<\! u + \tau / 2, \right.\\ &&\qquad\qquad\qquad\qquad \left. t^{rep}_{i} \ne t^{rip}_{j}\right\}, \end{array} $$

where $t^{rep}_{i}, t^{rip}_{j}$ are times of replay events and SWR events respectively (thus, for positive intervals $t^{rep}_{i} - t^{rip}_{j}$ the SWR event occurs first), and *Tδt* is the observed duration of the two processes, in seconds. The discretisation parameter *δt* of our model and the average duration of SWR events determine the minimum discernable lag between replay and SWR events, and thus our choice of *τ*.

We compare $\hat {\rho }_{rep, rip} \left (u \right )$ at various lags *u* with the theoretical value of Eq. (29) for unrelated processes, estimated by $N_{rep} \left (T \delta t \right ) N_{rip} \left (T \delta t \right ) / \left (T \delta t \right )^ 2$, where $N_{a} \left (t \right )$ is the number of events of point process *a* in the interval (0, *t*]. $\hat {\rho }_{rep, rip} \left (u \right )$ being greater than this for lags close to zero signifies that events of the processes occur at approximately the same time.

Brillinger (1976) shows that, for $T \delta t \to \infty $, for each *u* separated by *τ*, the $J_{rep, rip} \left (u \right )$ follow independent Poisson distributions with parameter $T \delta t \tau \rho _{rep, rip} \left (u \right )$. The dependence of the estimator distribution on the parameter being estimated suggests a variance-stabilising square root transformation. Thus, independently for each *u*, $\sqrt { \hat {\rho }_{rep, rip} \left (u \right ) }$ is approximately distributed as $\texttt {N} \left (\sqrt { \rho _{rep, rip} \left (u \right ) }, \left (4 T \delta t \tau \right )^{-1} \right )$. We use this fact to construct $\left (1 - \alpha \right ) \%$ confidence intervals around the estimates. We adjust the significance level *α* to account for our making multiple comparisons (one at each lag *u*) using the Bonferroni correction, which is to divide *α* by the number of comparisons made. This is very conservative as we are only interested in lags close to zero.

### Data simulation

We used simulated data (spike trains and position trajectory) to evaluate our parameter inference algorithm and our replay detection algorithm. The general simulation method, in which the parameterisation *𝜃*, *κ* is prespecified and data randomly simulated from the model with this parameterisation, is explained in Section 2.7.1. Section 2.7.2 explains how we simulate a set of spike trains in which multiple instances of a trajectory segment are encoded for the purpose of evaluating our replay detection algorithm.

#### Simulation of observation processes

For the evaluation of our parameter inference algorithm, we used a known parameterisation of the model to simulate spike trains and positions from we which made estimates of the parameters to compare with the known values. We first specified a model size *κ* ^∗^, then used an initial run of the algorithm of Appendix C with fixed state space dimension *κ* ^∗^ on the experiment data to find a set of realistic parameter values *𝜃* ^∗^. Then we sampled a sequence *s*_0:*T*_ by setting *s*_0_ to 1 (an arbitrary choice), then sampling *s*_*t*_ from the discrete distribution $\mathbf {P}^{*}_{s_{t - 1}, \cdot }$ for $t \in \left \{1, 2, \dots , T \right \} $. Positions and spike trains were then generated, on the discrete time scale, by sampling *x*_*t*_ from the distribution with probabilities $p \left (X_{t} = x \mid S = s_{t}, \theta ^{*} \right )$, and *y*_*t*, *n*_ from $\texttt {Poi} \left (\lambda ^{*}_{s_{t}, n} \right )$ for $n \in \left \{ 1, 2, \dots , C \right \}$.

#### Replay simulation

We assessed our replay detection algorithm of Section 2.5 by applying it to simulated spike train data in which known replay events were inserted. Our approach was to generate spike trains that correlate (via our model) with a random hidden position trajectory punctuated by instances of the template trajectories discussed in Section 2.5.2.

To achieve this we fixed *𝜃* ^∗^, *κ* ^∗^ as in Section 2.7.1 and simulated a full trajectory *x*_1:*T*_. Then, for each of several templates $x_{1:a_{r}}^{(r)}$, we selected uniformly at random *N*_*r*_ time bins between 1 and *T*−*a*_*r*_+1 as the replay event times, and at each event time *u*, we set $x_{u:u + a_{r} - 1} \gets x_{1:a_{r}}^{(r)}$. No two events were permitted to overlap: we resampled the later event time whenever this occurred. We then used the forward-backward algorithm to compute the smoothing posteriors for the state process *S*_1:*T*_ using the position trajectory alone, and used these to compute the posterior mean firing rate

32 $$ \bar{\lambda}_{n} = \sum\limits_{i = 1}^{\kappa^{*}} \lambda_{i, n} p \left(S_{t} = i \mid x_{1:T}, \theta^{*} \right) $$

for each cell *n*, at each time step *t*, then sampled a number of spikes for cell *n* in time bin *t* according to the Poisson distribution with mean $\bar {\lambda }_{n}$.

## Results

### Parameter and model size estimation

#### Simulated data

Using the method of Section 2.7.1, we simulated two data sets, distinguished by the domain used for position variables: one each corresponding to the linear track environment and the T-maze. In the simulated linear track data we used *C* = 4 and *κ* ^∗^ = 4, and in the simulated T-maze data we used *C* = 10 and *κ* ^∗^ = 5. This data we supplied to our model fitting algorithm to obtain estimates $\hat {\theta }, \hat {\kappa }$.

Using a flat prior over the number of states up to a maximum value of 10, our algorithm correctly identified *κ* ^∗^ in both data sets, using the modal value of *K*_*T*_ as explained in Section 2.4.2. Increasing the maximum number of states beyond 10, we found negligible posterior probability for larger models. In Tables 1 and 2 (corresponding to the linear track data and the T-maze data respectively) are measures of accuracy for our estimates of the conditional distributions over position given state and for rows of the transition matrix, by means of the Kullback-Leibler (K-L) divergence from a target distribution to the estimated distribution. The K-L divergence (cf. Dayan and Abbott (2001), p323) is a nonsymmetric distance between distributions; it has a minimum of 0, which is obtained if and only if the distributions are identical. In these tables we compare the K-L divergence from each target distribution to our estimates, against the K-L divergence from the target to a uniform distribution on the same support. The uniform distribution represents an estimate based on no data. We find that the K-L divergences from the targets to our estimates is one or two orders of magnitude smaller than those to the uniform distribution for each position model, and four or more orders of magnitude smaller for each row of the transition matrix, suggesting good accuracy for our estimates.

**Table 1:** Performance of model fitting algorithm: K-L divergence (in bits) of estimated model distributions, conditional on state, from target (simulated) distributions

State	Position model		Transition matrix row	
	Estimated	(Uniform)	Estimated	(Uniform)
1	5.39×10^−2^	1.78	8.17×10^−5^	1.95
2	1.54×10^−2^	1.48	4.34×10^−4^	1.75
3	1.16×10^−1^	1.39	4.55×10^−4^	1.77
4	1.57×10^−1^	1.82	5.51×10^−4^	1.95

Divergences of uniform distributions of appropriate size are provided for comparison. *Data set 1: simulated linear track*

**Table 2:** Kullback-Leibler divergences (in bits) of estimated model distributions from true values, as in Table 1

State	Position model		Transition matrix row	
	Estimated	(Uniform)	Estimated	(Uniform)
1	3.80×10^−1^	3.18	9.74×10^−4^	2.29
2	1.96×10^−1^	2.17	3.89×10^−3^	2.26
3	2.73×10^−1^	2.02	9.67×10^−4^	2.29
4	2.86×10^−1^	1.83	1.62×10^−3^	2.28
5	1.01×10^−1^	2.55	2.90×10^−3^	2.29

*Data set 2: simulated T-maze*

We used *H* = 1000 particles. Increasing this number was found not to significantly change the results. In preliminary runs, we found that it was necessary to scale up *H* between linearly and quadratically with the length of the data record in order to keep the effective sample size (ESS) (Kong et al. 1994), given by

33 $$ ESS = \frac{H}{1 + var \left(w \right)} $$

where $var \left (w \right )$ is the sample variance of the weights, above 50 *%*.

#### Experimental data

We applied the algorithm of Section 2.4 to the linear track and the T-maze data sets, using the first half of RUN epochs with a discretisation bin width of *δt* = 100ms, and found $\hat {\kappa } = 7$ for the linear track and $\hat {\kappa } = 8$ for the T-maze. For this we used $\bar {\kappa } = 10$ (after some exploratory runs of the algorithm with greater $\bar {\kappa }$ to eliminate larger models and greater *δt* for faster computation) and *H* = 800 particles for the linear track data and *H* = 1,200 particles for the T-maze. Increasing the number of particles beyond these values was found not to significantly change the output.

**Fig. 3:**
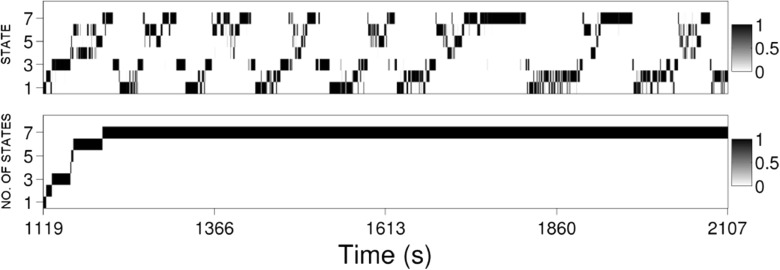
Marginals of the estimated smoothed posterior distribution over the state and number of states of the augmented model at each time step, Eq. (23), in the linear track data with *δt* = 0.1s

The smoothed posterior distributions over augmented states Eq. (23) are depicted for each time step in Fig. 3. The marginal distributions over state *S*_*t*_ and number of states observed *K*_*t*_ are presented separately. In the former, it can be seen how intervals of data are very unambiguously labelled by state: the distribution at each time step is highly concentrated. The latter is used to estimate the number of states required to characterise the data; the interpretation in this case is that after taking all of the training data into account, the most probable number of states between 1 and $\bar {\kappa }$ is 7. In these runs the resample-move procedure was found to be effective in rejuvenating the sample with the ESS, Eq. (33), typically over 50 *%*.

**Fig. 4:**
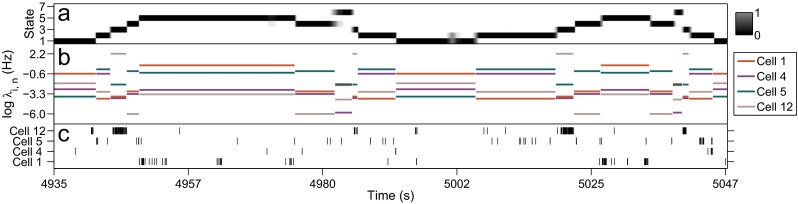
Segment of the T-maze RUN data exhibiting the model characterisation of spike trains. **a** smoothing posterior distribution over hidden state at each time step. **b** mean spike rate (in log domain for clarity) conditional on the MAP hidden state for four cells in the sample. **c** rasters of observed spike times

**Fig. 5:**
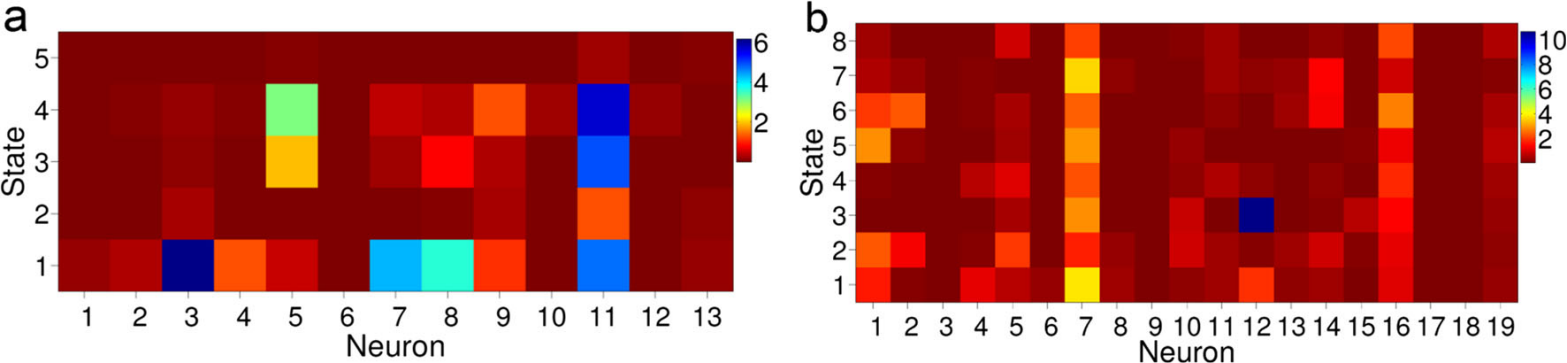
Mean spike rates, $\hat {\lambda }_{i, n}$, in spikes per second for states $i = 1, 2, \dots , \hat {\kappa }$ and neurons $n = 1, 2, \dots , C$. **a** linear track. **b** T-maze

Figure 4 depicts, for an interval of T-maze RUN data, the smoothing posteriors over the hidden states *S*_*t*_ and how the changing state corresponds to changing levels of activity in the spike trains. The middle panel of the figure shows, for several cells *n*, the value of $\log \hat {\lambda }_{\hat {s}_{t}, n}$, with $\hat {s}_{t}$ the MAP state at time *t*, as a piecewise continuous line. By comparing these jumping spike rates to the spike trains represented by the raster plot in the bottom panel, one can see how different states correspond to different levels of cell activity and how the Markov chain characterises variability in the activity of all cells simultaneously. All spike rate estimates are plotted as a heat map in Fig. 5.

**Fig. 6:**
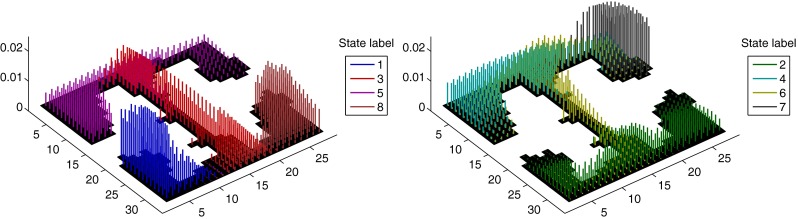
Model characterisation of position in T-maze data: each cluster of vertical bars of a single colour represents the distribution over the discrete positions on which they stand, conditional upon a particular state. The height of each bar indicates probability mass. The units on the x- and y-axes and bin indices - each bin was approximately 5cm square

Figure 6 depicts the estimated distributions over positions conditional on state for the T-maze data. Probabilities are represented by the height of bars and states are distinguished with different colours. These demonstrate how the states of the Markov chain constitute a coarse-grained representation of position: broad regions of the environment are associated with a particular state, characterised by a central position and covariance structure.

### Position decoding

This section compares the performance of our model with two other models previously used for decoding: the BD model, as explained in Zhang et al. (1998), and the LP HMM. Our implementation of these models is described in Appendix G. In BD and LP, positions *X*_*t*_ are used as states (instead of our *S*_*t*_ variables) with state space of size *M*, and in LP (following Johnson and Redish (2007)), a transition matrix with rows constrained by Gaussian distributions centered on each position. The spike count in each time bin is modelled as a Poisson random variable in each model (conditionally on position in BD and LP, conditionally on state in OP) for fair comparison (a Bernoulli model was used in Johnson and Redish (2007)). Maximum likelihood is used for parameter estimation in each model. See Appendix G for further details about estimation using the BD and LP models.

For these results we used the second half of RUN data, i.e. distinct from that used for parameter estimation (*cross-validation*), and we used the T-maze data since it presents more of a challenge for decoding due to its corners and larger size. We use our fitted model with $\hat {\theta }, \hat {\kappa }$ and the approach to decoding explained in Section 2.4.3.

#### Decoding comparison: data and performance measures

**Fig. 7:**
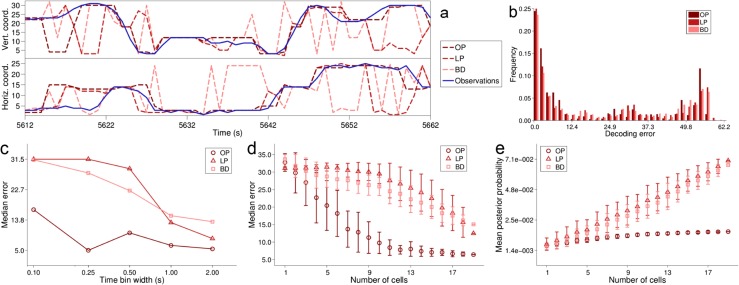
Comparison of position decoding performance under our model (OP), the HMM of Johnson and Redish (2007) (LP) and the model used in Zhang et al. (1998) (BD). **a** Viterbi estimates of position under each model alongside the observed trajectory (*blue*) in a segment of the T-maze RUN data; *δt* = 1s, *C* = 19. **b** empirical distributions of decoding errors (distance between estimated and observed position; units are spatial discretisation bins, which have length ∼5cm) for the second half of the T-maze RUN data for the three methods; *δt* = 1s, *C* = 19. **c** Median decoding error found in same data for a range of values of the temporal bin width *δt*. **d** Median decoding error found when subsets of the cell sample were used in decoding. Error bars indicate 1 standard deviation either side of the mean for the subsets used. **e** for the same subsets of cells, the mean of the probabilities of the observed position at each time step under the smoothing posteriors computed with each model

We used two measures of performance: median decoding error and mean marginal posterior probability, and we used the same data (same spatial resolution) for each model. The decoding error of estimate $\hat {x}_{t}$ we defined as $\bar {d} \left (x_{t}, \hat {x}_{t} \right )$ (the distance function of Section 2.2.3). We then took the median of the decoding errors over all *t* (rather than the mean since the mean was affected by the heavy tail of the error distribution for all three methods, as shown in Fig. 7).

The mean smoothed posterior probability of *x*_1:*T*_ is

34 $$ \frac{1}{T} \sum\limits_{t = 1}^{T} p \left(x_{t} \mid \mathbf{y}_{1:T}, \hat{\theta} \right), $$

where each term in the sum can be computed with the algorithm in Appendix E for our model, or with the forward-backward algorithm for LP. In BD these terms are the single time step posterior probabilities. For an accurate model, the observed trajectory will pass through regions of high posterior probability. Thus, since greater posterior probability on particular positions reduces the posterior variance, a greater value for this measure indicates confidence as well as accuracy, on average, for the decoding method.

#### Decoding comparison: results

As per Section 2.4.3, we used the Viterbi algorithm to decode position as the most probable path given all spike train observations. This is the standard Viterbi algorithm (Viterbi 1967) for an HMM for LP, and the algorithm of Appendix D for OP. For BD the Viterbi estimates are simply the maximum likelihood estimates. A typical interval of the test data is plotted in Fig. 7, *top left*, showing each set of decoded estimates alongside observations. The BD estimates have a tendency to jump erratically, whereas the estimates obtained with the HMMs are smoother. Also visible in this figure, towards the end of the interval, is the tendency for the LP estimates to become trapped around one erroneous estimate. This is particularly a problem for small *δt* when it results in massive decoding errors.

For each method we computed the performance measures described in Section 3.2.1 using models fitted under different values of the parameters *δt* and *C*. For each value of *C* less than the total number of cells available, $C^{\max }$, we had a choice of population subset to use; we computed the measures on each subset in a sample of 100 selected at random, or $\left (\overset {C^{\max }}{C}\right )$ if $\left (\overset {C^{\max }}{C}\right ) < 100$. Each model was re-fit (i.e. SMC parameter inference of *𝜃* including *κ* for OP) for each value of *δt* considered; for each value of *C* it was only necessary to ignore neuron labels not in the sample.

These results are presented in Fig. 7, *bottom row*. In the bottom left figure is shown how the decoding performance of OP, as quantified by the median error, does not deteriorate drastically with increasing temporal resolution over the range of values of *δt* considered (2s, 1s, 0.5s, 0.25s and 0.1s), unlike LP and BD. The ability of these latter models to decode accurately is severely impaired for *δt*≤0.5s. The median error of decoding and mean posterior probability for varying *C* are plotted in the bottom centre and bottom right plots, respectively. For these results we fixed *δt* = 1s. The error bars in these plots indicate one standard deviation either side of the mean for the cell subsets corresponding to each *C*. We see that in both measures the decoding performance of OP does not degrade much until *C* is reduced to about 6 cells, but the performance of LP and BD is badly affected by decreasing *C*. The mean posterior probability of OP is generally lower than for the other models because the posterior variance over positions is generally greater; this is because we do not model positions individually but via a small number of conditional distributions with inherent uncertainty (cf. the position model in Section 2.2.3).

The distribution of decoding errors using estimates obtained with each model, and with *δt* = 1s and *C* = 19, is plotted in Fig. 7, *top right*. This shows that all three methods suffered from long range errors, but OP did not suffer the very worst errors and had a greater proportion of short range errors than LP and BD. These long range errors are caused by a tendency, in each model, to decode particular positions during times of low firing rates; this is discussed further in Section 4.2.

### Replay analysis results

#### Simulated data

To assess the performance of our replay detection method on simulated data, we considered replay detection as a binary classification problem where each time bin is to be classified as participating in a replay event or not. First we simulated a REST data set consisting only of spike trains, with 40 known replay events (20 from each of 2 short templates) using the method explained in Section 2.7.2. Then, using $\hat {\theta }, \hat {\kappa }$ estimated on the simulated RUN data set (discussed in Section 3.1.1), we applied our replay detection algorithm of Section 2.5 with a range of values for Ω^∗^, and computed the receiver operating characteristic (ROC) curve parameterised by Ω^∗^.

Since the ROC curve does not take the rate of false negative classifications into consideration, we also looked at the Jaccard index (Pang-Ning et al. (2006), p74) as an alternative classification measure at each Ω^∗^ considered. Let *TP* and *FP* be respectively the number of true and false positive classifications and let *FN* be the number of false negative classifications, then the Jaccard index is

35 $$ J \left(\Omega^{*} \right) = \frac{TP}{TP + FP + FN}. $$

The maximum value of 1 can only be attained when *FN* = 0, i.e. when no true replay time bins have been misclassified. Thus, the Jaccard index complements the ROC curve by taking into consideration any failure of the algorithm to detect a replay event.

**Fig. 8:**
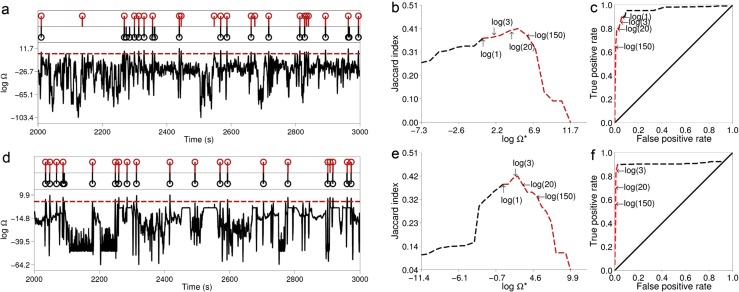
Evaluation of replay discovery in simulated data. **a** black trace is replay score for a template (on the simulated linear track), plotted at the midpoint of the template as it is moved across the data. The red line indicates a threshold of Ω^∗^ = 20. Red stems indicate times of replay discovery (when a local maximum of the replay score exceeds Ω^∗^); black stems indicate times of replay events simulated using the method described in Section 2.7.2. **b** and **c** respectively the Jaccard index curve and ROC curve for discovery of replay of the template considered as binary classification. In each plot the curve is parameterised by Ω^∗^; the red segment corresponds to Ω^∗^>=1. **d**-**f** similar plots but for a particular template in the simulated T-maze data set

The ROC curve and Jaccard index for the replay detection of one template in each of the simulated data sets are presented in Fig. 8. In both data sets the false positive rate is low (<5 *%*) for Ω^∗^>1, with good true positive rates (>70 *%*) for a wide range of Ω^∗^, and is still about 60 *%* for the conservative Ω^∗^ = 150. The Jaccard index reaches a peak for positive Ω^∗^ in this range and only starts to decrease beyond Ω^∗^ = 150. Also in Fig. 8 are plotted the corresponding profiles of Ω (as a logarithm, for clarity) and the times of simulated and detected replay for a particular value of Ω^∗^. It can be seen how the times of replay detection (red stem markers) refer to the times of maxima of Ω above the threshold. In both data sets most of the replay events are discovered (97.5 % in the linear track, 75 % in the T-maze) with a small number of false positive errors.

**Fig. 9:**
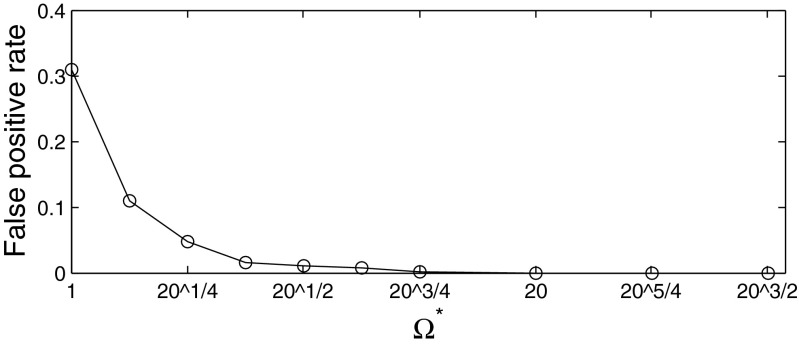
False-positive rate against Ω^∗^ in the case of mis-specified templates

We also conducted experiments to investigate the frequency of false-positives in the case of mis-specified templates, meaning the evaluation of Ω for templates which were *not* either of those used in the generation of the data, . In this situation, there is never a ‘true positive’, so the ROC curve is not an appropriate way to display performance. Instead, in Figure 9, we simply plot the rate of false positives against the threshold parameter Ω^∗^. The result here are for the T-maze. From this curve, we can see that the rate of false positives drops rapidly to zero as Ω^∗^ increases, indicating that the method correctly recognizes the template in question is not consistent with the data. Similar results were obtained for the liner maze (not shown).

#### Replay in experimental data

**Fig. 10:**
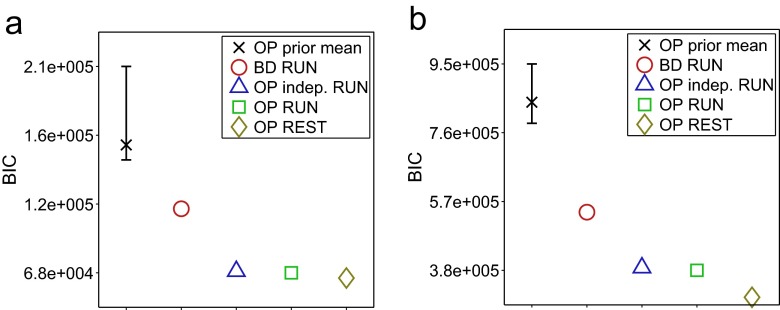
Bayesian information criteria (BIC) for model fit assessment on the REST data, used for replay analysis. The green square represents our model (OP) with parameter values fitted to RUN data. This we compare against: parameterisations of the OP sampled from the prior for *𝜃* (error bars indicate the 5th and 95th percentiles of the sample) and the expectation of the BIC over the prior, the Bayesian decoder fitted to RUN, the OP fitted to RUN but with its Markov chain dynamics replaced with a time invariant distribution over states, and the OP fitted directly to the REST data. **a** linear track data, **b** T-maze data

We applied the algorithm of Section 2.5 to our experimental REST data sets using $\hat {\theta }$ estimated from RUN data. First we used the model comparison approach described in Section 2.5.4 to verify that the model with parameter values $\hat {\mathbf {\theta }}$ was a good fit to the REST data in both data sets. As shown in Fig. 10, the BIC on the REST data for our model with $\hat {\theta }$ estimated from RUN data (OP, RUN, *green square*) is close to the benchmark parameterisation - the model fitted to the REST data directly (OP, REST, *gold diamond*) - relative to the model with *𝜃* sampled from the prior and the prior mean (*black cross*). We draw reassurance from this that the model with parameterisation $\hat {\theta }$ learned from RUN is a good fit to the REST data used for the replay analysis.

This is further supported by OP (RUN) having a lower BIC than the similar model parameterisation with independent rather than Markovian dynamics (Section 2.5.4), also shown in Fig. 10. Thus, the dynamics from RUN, as characterised by $\hat {\mathbf {P}}$, persist in REST and are described well by $\hat {\mathbf {P}}$. We also compare the BIC on REST data of OP (RUN) with that of BD, fit to RUN. We find that the former is much lower, both with and without Markovian dynamics, implying that with its smaller state space, our model is a more parsimonious characterisation of the data. Moreover, this reassures us that a model including a characterisation of awake behaviour-related dynamics is appropriate for use on REST data, which has been a concern for authors such as Davidson et al. (2009) who opted against such models for this reason.

**Fig. 11:**
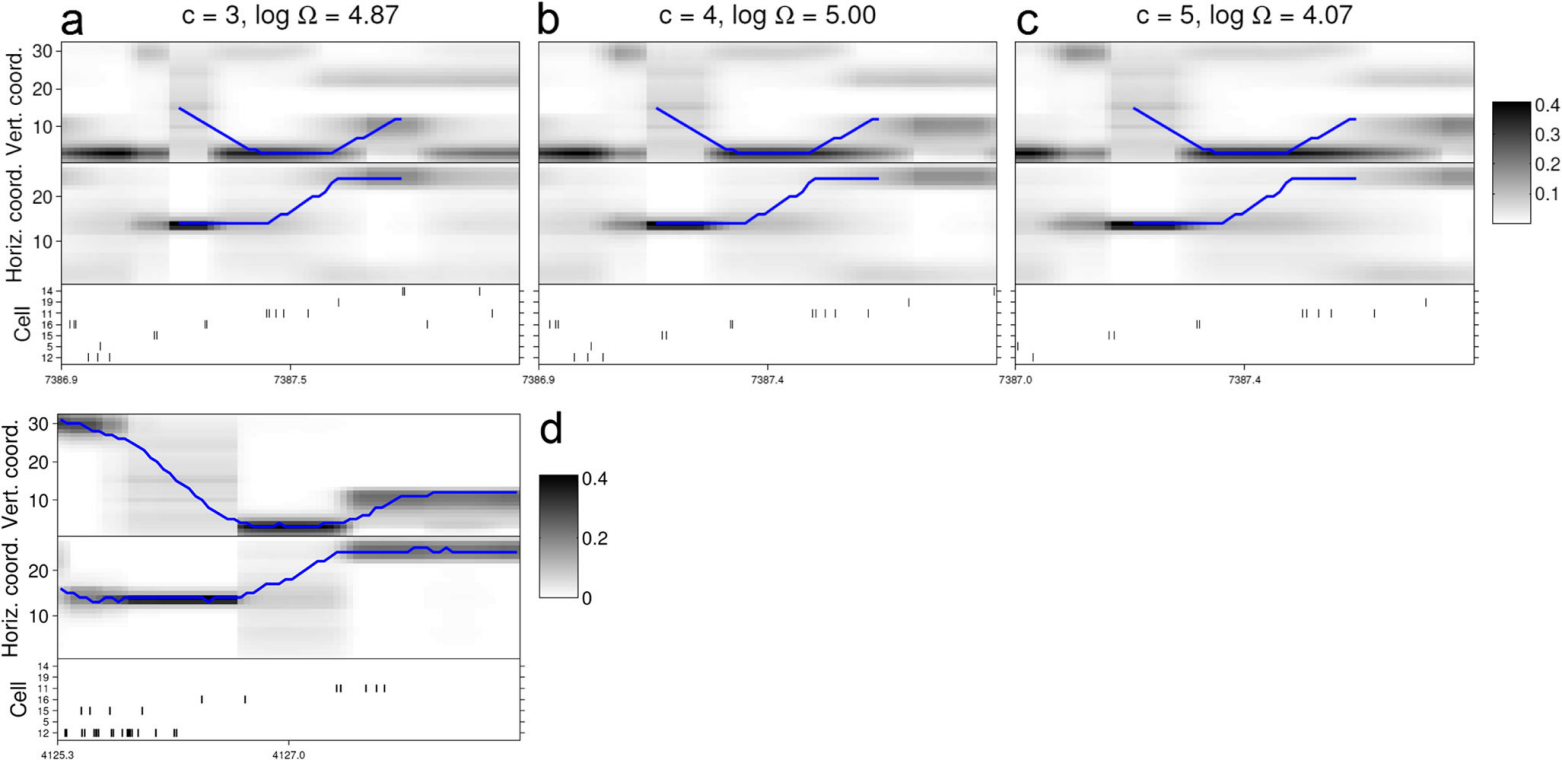
Example of a replay event discovered in experimental data. Each subfigure depicts a time interval around a discovered replay event. In the top two panels are plotted, at each time step, the smoothing posteriors over position (obtained using Eq. (24)), marginalised to the vertical and horizontal position coordinates, with a greyscale shade indicating probability. A blue line indicates the template trajectory. The bottom panels depict a subset of the concurrent spike trains as a raster of spike times: only cells that spiked during the interval are represented. **a**-**c** the same replay event as discovered in the T-maze REST data at compression rates 3, 4 and 5; the peak replay score was observed for this event at a compression rate of 4. **d** a similar interval around a discovery of the same template in the T-maze RUN data. Here the blue line describes the observed trajectory

In order to demonstrate more explicitly how our replay detection works, in Fig. 11 is depicted an example of a detected replay event of a template in the T-maze data. This template comprises a path around the forced turn and into a rest area. The images depict the smoothed posterior distributions over position (with greyscale shade indicating probability mass), marginally for the two spatial dimensions, at each time step in an interval around the event. The top row of the figure shows the replay event at three consecutive compression rates *c*, with the central panel showing the event detected with peak Ω at compression rate *c* = 4. Also plotted is the template trajectory, in blue, and a raster of spike times for all cells that spiked during the interval. Regions of high posterior probability follow the template, and greater Ω corresponds to a closer fit of the template to the position posteriors. Below and to the left of the figure is plotted an example of the same template being matched against an interval of uncompressed RUN data, now with the observed trajectory depicted in blue. We see a similar trajectory of peak posterior probability tracking the observed trajectory, which gives us confirmation (by eye) that the episode detected in REST matches an encoded RUN experience. We also see in this interval of RUN a similar pattern of spike trains from the same cells as in the replay event.

Details of our replay analysis are summarised in Table 3. Using a threshold of Ω^∗^ = 20, we found 326 and 1,398 events in the linear track and T-maze data sets respectively.

**Table 3:** Summary of REST data sets used for replay analysis and results

Data set	*δt* (s)	*C*	*T* ^RUN^	*T* ^REST^	*κ*	$\hat {\kappa }$	#Templates	Mean template duration (s)	#Replay events	#SWR events	Mean SWR duration (s)
Sim. linear track	0.1	4	10,000	10,000	4	4	2	4.80	39 (of 40)	n/a	n/a
Sim. T-maze	0.1	10	10,000	10,000	5	5	2	2.50	30 (of 40)	n/a	n/a
Linear Track	0.1	13	9,879	9,708	n/a	7	23	4.26	326	261	0.07
T-maze	0.1	19	22,569	39,943	n/a	8	309	2.54	1,398	1,492	0.07

### Correlation of replay events with hippocampal SWRs

**Fig. 12:**
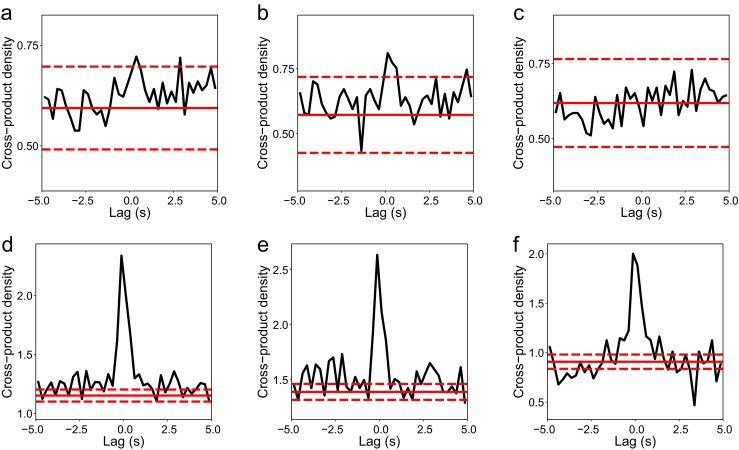
Estimates of the cross-product density $\sqrt {\rho _{rep, rip} \left (u \right )}$ from times of SWR events to times of detected replay events at lags *u* around 0, obtained from the cross correlogram, $J_{rep, rip} \left (u \right )$. Estimates have been square root transformed for variance stabilisation, as in Brillinger (1976). Solid red lines indicate $\sqrt {\rho _{rep} \rho _{rip}}$, the value expected for two independent processes. Dashed red lines indicate approximate confidence limits constructed using a significance level of *α* = 0.178 *%*, which includes the Bonferroni correction for comparing the estimate at each lag. **a**-**c** linear track, **d**-**f** T-maze. **a** and **d**, all REST data used; **b** and **e** first half of data; **c** and **f**, second half of data

We used the methods described in Section 2.6 to identify SWR events in the LFP recorded during REST for each data set (summarised in Table 3). We computed the cross correlogram for the times of SWR events and replay events, using a bin width of *τ* = 0.25s, appropriate to the *δt* used and the average duration of SWR events. As explained in Section 2.6, this is an unbiased estimator of the second-order product density function, $\rho _{rep, rip} \left (u \right )$. Values of $\sqrt { \hat {\rho }_{rep, rip} \left (u \right ) }$ are plotted in Fig. 12, between −5s and 5s. An approximate 0.178 *%* confidence interval, which includes a Bonferroni correction for multiple comparisons, is plotted around the value for $\rho _{rep, rip} \left (u \right )$ under the assumption of no correlation, to highlight deviations from it as peaks or troughs outside of the interval. The interval is wider for the linear track results because there fewer events were detected (likely due to shorter recordings, i.e. smaller *T*).

We observe a significant peak around zero for both the linear track and T-maze data sets (Fig. 12, *left column*), from which we conclude that the times of replay events and SWR events coincide. The peak around zero extends into positive lags more than negative lags, signifying that the SWR events occur first (cf. Eq. (31)) as would be expected if replay occurs during ripples. Regarding peaks away from zero we must consider that estimates of $\rho _{rep, rip} \left (u \right )$ become less reliable as the lag |*u*| increases (Brillinger 1976). The results presented in Fig. 12 were based on the replay events detected using a threshold of Ω^∗^ = 20. Using the more conservative threshold of Ω^∗^ = 150 we draw the same conclusions, except in the case of the linear track data for which we did not have enough events to demonstrate a significant correlation.

We defined the second-order product density function for *stationary* processes. In order to guard against deviations from stationarity affecting our results, we performed the same analyses on events detected in subsections of REST. These are plotted in Fig. 12, *middle* and *right*. We find that the correlation between the processes persists at this finer scale in the T-maze data. No significant correlation is found in the second half of the linear track data, but the correlation does exist in the first half, so the correlation does persist across different scales in at least part of the data.

**Fig. 13:**
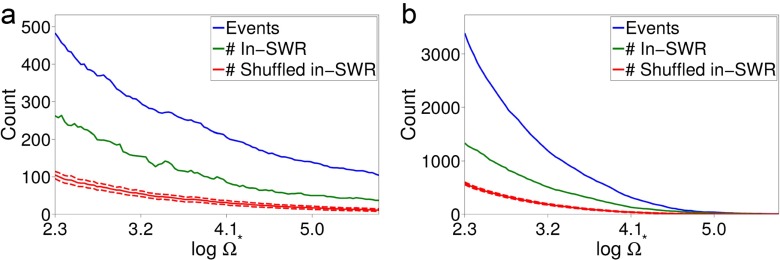
Relation of replay threshold Ω^∗^ to number of replay events detected and number coincident with SWR events. The blue line is the number of events detected using the approach summarised in Section 2.5.5, the green line is the number of events that coincide with SWRs (at least 50 *%* temporal overlap), and the red line is the mean number of coincidences of replay events after they have been shuffled 500 times (dashed line indicates one standard deviation either side of mean). Values of Ω^∗^ at 200 points evenly distributed between Ω^∗^ = 10 and Ω^∗^ = 300 were used. **a** linear track data, **b** T-maze data

Figure 13 shows how detected replay events match with SWR events at different levels of replay threshold Ω^∗^. We identified coincidence of replay with an SWR when the extent of a (temporally compressed) replay event overlapped with an SWR by at least 50 *%*. The proportion of events coincident with an SWR (in-SWR events) was about half of all events over every value of Ω^∗^ considered. To account for chance coincidences, at every value of Ω^∗^, we shuffled replay event times uniformly at random and recalculated the number of coincidences. The mean of 500 shuffles, and one standard deviation either side of the mean, is also shown in Fig. 13. The observed coincidence rates are far greater than the shuffled sample at each value of Ω^∗^, which, in light of the known association of replay with SWRs, attests to the accuracy of our methods. That the rate of coincidences is approximately constant with respect to Ω^∗^ suggests that the rate of false positives is low. This is because false positives are equally likely to be made outside of SWRs as inside, and the time inside SWRs is a small proportion of the REST data; thus if there are many false positives, as Ω^∗^ is increased and false positives are eliminated, the coincidence rate should increase.

## Discussion

### Improvements afforded by our model

In developing our model, we recognised the advantages of the statistical modelling approach to spike train analysis: that sources of variation in observation variables are explicitly accounted for, enabling one to quantify the probability of outcomes and make predictions. Furthermore, we recognised the advantage of including dynamics via the HMM framework, as undertaken by Brown et al. (1998) and used for replay analysis in Johnson and Redish (2007), for the accurate characterisation of data with clear dependence through time.

By removing position observations from the hidden process - the approach of LP - out to an observed process parallel to the spike trains (cf. Fig. 1), we achieve two important improvements. Firstly, we elicit from the data itself structure around the trajectory of the animal and how this relates to the spike trains, within the constraints imposed by our model distributions. This structure is described by the number, location and shape of broad regions of the environment that are, to the extent permitted by the data, the smallest regions discernable by variation in the spike trains. We bring to this inference no prior knowledge, using uninformative priors as far as possible, including our inference for the number of states, thus allowing the data to “speak for itself”.

Secondly, the disassociation of discrete positions from states of the model, which reduces the number of parameters to the small set necessary for our coarse-grained representation of space. This parsimony is confirmed by the lower BIC for OP than BD (cf. Fig. 10). This makes it easier to make robust estimates of the parameters with limited data, and, by performing inference for the number of states and for the position model parameters, we are able to explore the neuronal ensemble’s representation of space via the number, size and shape of these regions; this is demonstrated in Fig. 6. Further opportunities are provided for studying the brain’s representation of space by performing these inferences under different experimental conditions, such as different stages of the animal’s training or familiarity with the environment.

### Decoding performance of the model

There are two important consequences for our model of the decoding analysis presented in Section 3.2. Firstly that the catastrophic rate of decoding error we observe with BD and LP at high temporal resolution does not occur with OP. This means we are able to use a greater resolution at the parameter estimation stage and thereby capture variations in the spike count that occur on a more precise time scale.

The reason for this benefit seems to be OP’s coarse-graining of position to a small number of minimally discernable regions. The problem seen in BD and LP has to do with an unwanted feature of these kinds of model: that it implies some positions are encoded by the absence of spikes. Zhang et al. (1998) noted decoding errors in the form of large jumps or discontinuities in decoded trajectories, mostly occurring when the animal was still and firing rates were low. It appears from their Fig. 3 that these erroneous decoded estimates were of a small number of particular positions. This has also been our experience using these methods, in particular at high time resolution, as exhibited by the jumps in decoded trajectory in the top left plot of Fig. 7. We have also observed trajectories decoded using LP getting trapped in particular locations at high time resolution (*δt*<1s). In both BD and LP, particular positions maximise the likelihood (conditional probability of spike train observations given position) for low spike counts, and so will maximise, or at least strengthen, the posterior distribution over these positions, and hence they will be decoded with methods based on the likelihood.

In OP, however, a particular state will maximise the likelihood for low spike count observations, but these periods are brief relative to the jump rate of the Markov chain due to the relatively small number of states, and so these observations will not have such an overpowering effect on the posterior. Thus, the consequence of positions encoding inactivity are avoided in OP by its association of broad regions, rather than discrete positions, with states of the model, and by eliciting the details of these regions from the data itself.

The second advantage conferred by OP as demonstrated by our decoding results is that we can achieve good results with a small number of cells (little degradation in decoding performance for a sample of 6 or 7 cells compared with 19 cells). This makes our model a good choice for decoding with limited data, as may be the case when we wish to record from a particularly idiosyncratic or sparse population of neurons, when recordings are of poor quality and cannot be easily clustered, or when less advanced equipment is available.

More than simply as a tool for inferring the information content encoded in spike trains, decoding using the posterior distribution (including Viterbi estimates) can be seen as a posterior predictive validation of the model (Gelman et al. (2003), p188); that is, as a means for veryifying the statistical model’s characterisation of the encoding of position in spike trains. The decoding results thus support our model as being useful for the study of replay, and, since our method for replay detection is based on the same principle as the decoding algorithm - that of using the posterior distribution over position to infer the information content of spike trains - the advantages demonstrated for our model in decoding also apply to replay detection.

The decoding results presented here demonstrate a relative performance benefit of OP over BD and LP. The results presented in Fig. 7 do not compare well with other studies focussing on decoding, e.g. Brown et al. (1998), but these tend to make use of additional information that our model would also accommodate. For example, we could include additional covariates alongside spike trains and position, such as phase of the theta rhythm (as do Brown et al. 1998). Also, whilst we used all of (the first half of) a RUN epoch for parameter inference, we could use prior knowledge that the activity of place cells depends on behavioural state, in particular they have more robust spatial selectivity during movement and replay non-local information during pauses in exploration, by using only the subset of RUN data in which the animal was in motion for model fitting (e.g. Pfeiffer and Foster 2013).

Decoding performance could be improved by partitioning training data by behavioural state, and only using the subset of data in which the animal was in motion, because of the presence of awake replay during pauses in exploration. The effect of awake replay, if included in training data, would be that spike train activity is associated with multiple positions: the “local” spike activity and the ”nonlocal” activity. This would in particular have an adverse effect on the parameters of BD and LP (the spike rate map, Eq. (69)) because it relates specific activity to specific positions; the effect on OP would be less severe since its states are associated with global spike train patterns. States of OP are not identified with positions, as in BD and LP, so the interpretation of nonlocal activity in OP is more consistent than in those other models; thus, the observation of certain states at particular times, e.g. in the smoothed posteriors exhibited in Fig. 3, could identify different behavioural states or awake replay, a possible avenue for future work.

### The SMC algorithm

Other solutions to the identifiability problem in HMMs have been proposed, but these come with their own issues. As discussed in Scott (2002), these often involve imposing structure on the prior distribution of exchangeable parameters, or otherwise breaking the symmetry in the model. This kind of solution is difficult to justify when there is no a priori reason to bias parameters away from each other or impose constraints on, for example, the ordering of parameters such as mean firing rates, and inferences may be influenced by the choice of constraint. The solution presented in Chopin (2007) and used here does not require any such constraints, permits a fully Bayesian approach to parameter inference with uninformative priors and facilitates a Bayesian approach to model selection that accomplishes the task of eliciting from the data itself the required complexity for the spatial representation. Inference for parameters is subject to sampling error, but targets the true values, unlike in the methods of Chen et al. (2012), and we can achieve an arbitrary degree of accuracy by increasing the particle sample size, constrained only by computer resources.

Inference for model size *κ* permits our model to achieve the parsimony discussed in Section 4.1. Our model partitions the observation processes into piecewise stationary intervals (i.e. homogeneous Poisson processes for spike trains) labelled by state of the hidden Markov chain; thus inference for *κ* targets the most efficient such partition into stationary intervals permitted by data. This is also constrained by the time discretisation, since, under smaller values of *δt*, more rapid spike train dynamics become accessible and must be accounted for. In Section 3.2 we compared different decoding approaches under different discretisations of the data; in this we did observe that our estimate for the number of states, $\hat {\kappa }$, increased with decreasing *δt*: $\hat {\kappa } = 6$ was found for *δt* = 2s; $\hat {\kappa } = 8$ was found for *δt* = 0.1s for the T-maze data set. In future work we may generalise the model to continuous time, with the intention of capturing spike train dynamics on all time scales.

There are several algorithmic parameters required to be set in the SMC algorithm, including the number of particles, the ESS threshold for resampling and the minimum size of a subpopulation to be maintained in resampling. Whilst increasing the number of particles decreases the Monte Carlo approximation error, and increasing the frequency of resample-move steps helps avoid inferior modes of the likelihood surface, both of these increase computational demand. There is thus a balance to be struck between precision of parameter estimates and computation time. The latter is also impacted by resolution parameter *δt*; for values *δt*<0.1s, computation time becomes excessive for useful parameterisations of the SMC algorithm. This is because, as discussed above, more states are exhibited in data with smaller *δt*, and computation time scales quadratically with *κ*. For greater precision, the generalisation of the model to continuous time, i.e. a Markov jump process, for which efficient MCMC inference procedures exist (e.g. Rao and Teh 2013). Computation time is most sensitive to *κ*; other parameters, such as *C* or *T*, have linear (or better) time complexity.

### Use of the BIC for model comparison on REST data

We chose to use a likelihood-based technique for model comparison, the BIC, to verify that the model fitted to RUN data was a good fit for the REST data. It was important for our application of replay detection using an evaluation of the posterior as in Eq. (25) that we assess the fit of a particular parameterisation of the model - the posterior mean estimate $\hat {\theta }$, in particular - rather than the model fit marginal of model parameters as is typically done in a Bayesian model comparison, for example with the deviance information criterion (DIC, cf. Gelman et al. (2003), p183) and the Bayes factor (Kass and Raftery 1995). Furthermore, our task was not merely to demonstrate the general out-of-sample predictive power of our model, as is achieved with the DIC, but predictive power specifically on the REST data. The BIC is useful for this because it can be computed using the REST data likelihood. The BIC also permits comparisons between non-nested models, for example between OP and BD, and its inclusion of a penalty for model complexity provides a stronger test for OP against the time-independent alternative (which has no transition matrix).

### Replay detection results

The numbers of replay events detected (Table 3) exceed those reported in other replay studies, meaning our methods provide more examples to study, increasing the power of analyses such as detecting cross correlation between events. For example, Ji and Wilson (2006) found about 39 candidate events (not restricted to those in SWRs) per session, Lee and Wilson (2002) found 57 events (based on triplet sequences of cell activation) between three rats, and Nádasdy et al. (1999) found up to 40 events (repeats of spike sequences) per session. These studies use spike sequence detection, which relies on precise spike timing and suffers low power (see, e.g. Naud et al. 2011), which our model-based approach does not on account of the generality of using posterior distributions over position given spikes to detect post-behaviour representations of activity. We also obtain more events than other model-based approaches, such as Davidson et al. (2009), who found on the order of 100 replay events per session. This can be attributed to our use of conditional distributions over position (cf. the replay score, Section 2.5.1) to explicitly account for variation over possibly-encoded trajectories; we therefore do not have to make the kind of restrictions these authors make on replay information content, such as to trajectories of constant velocity, to guard against spurious detection.

### Replay detection methods

The motivation for our approach to replay detection was to take further the model-based, decoding approach used profitably in other studies, and by so doing overcome the principle challenges associated with replay detection and enable a more extensive analysis of the phenomenon. Whereas studies such as Johnson and Redish (2007) have used the time marginal posterior distributions of position given spikes, discussed in Section 2.4.3, we use the posterior distribution over *trajectories*: sequences of position random variables considered jointly (cf. Eq. (25)). The neuronal representations we wish to identify in replay detection are dynamic: their temporal dependence structure is essential. It is therefore important for the detection of replay with a model of the relevant processes that one starts from the most general characterisation permitted, so one does not make any inappropriate assumptions (of independence, for example) that make the model itself appear inadequate. Indeed, we saw by comparison of the BIC in Fig. 10 that the model with temporal dependence between latent states was a better fit to spike train data than the same model with temporal independence.

In our method, the risk of mistaking chance observations for true replay is accounted for by the marginal distribution over trajectories, e.g. $p \left (X_{t} = x_{1}, \dots , X_{t + a - 1} = x_{a} \mid \mathbf {\theta } \right )$ for a template of length *a* at offset *t*; cf. Eq. (25). Setting a positive threshold for Ω protects against trajectories that may be probable a posteriori due to a bias in the model favouring those trajectories; we must have Ω>Ω^∗^ only when a trajectory is decoded above “chance” as represented by the marginal distribution. We do not need to resort to ad-hoc tests of statistical significance or the kind of shuffling procedures mentioned above, which have an element of subjective judgement in their design, nor do we need to accept any approximate *p*-values of uncertain accuracy.

### Appraisal of the template matching approach

For the results presented in Section 3.3.2 we used segments of an observed trajectory (i.e. from RUN data) as templates for replay detection. However, the decision of which segments to use was arbitrary, and was guided only by our interest in particular regions of the environment. This means we are unable to determine the true start and end times of a replay event, which also precludes us from drawing conclusions regarding the relationship between replay event duration and time compression rate. One possible approach to dealing with this issue is to develop a continuous time model in which the compression rate is instantiated as an unknown parameter to be inferred from data, which controls the overall rate of transitions between different latent states. Such an approach is likely to be more computationally demanding, however.

It may be possible to combine our replay analysis methods with the decoding algorithm to make a more comprehensive study of what is being replayed and at what compression that does not depend on our choice of templates, for instance by eliciting replayed trajectories directly from the data such as segments of the Viterbi path during REST.

Nevertheless, our template approach is very flexible and allows to control for the information content of replay. For example, we could limit our analysis to replay around an important maze feature, such as the choice T-junction in the T-maze. We could also use templates that represent trajectories in distinct environments (in particular, a different environment from the one the rat is in, (e.g. Gupta et al. 2010), or environments not yet visited (i.e. “preplay”, Dragoi and Tonegawa 2011).

## Conclusion

We have presented a dynamic statistical model relating multiple parallel spike trains to concurrent position observations that explains the data in terms of discrete levels of spiking activity and broad regions of an environment, corresponding to distinct states of a Markov chain. We have seen an improvement in decoding performance over other models which seem to be consequences of our use of states distinct from individual positions. In this way our model improves upon those of Brown et al. (1998) and Zhang et al. (1998), used in most recent studies of replay, in which positions are identified with states of the model. The approach taken to model fitting achieves Bayesian inference for parameters, overcoming the model identifiability problem suffered by HMMs with a likelihood invariant to permutations of the state, while also performing Bayesian inference for model size.

We have also presented a new model-based method for the analysis of replay in spike trains, and demonstrated how this can be employed with our model to discover replayed representations of position trajectories of arbitrary length and content. We have argued that consideration of the model likelihood, and how it compares with certain benchmarks, is an appropriate way to demonstrate a model as being an appropriate characterisation of data distinct from that used for parameter inference. Once this is established, our method for identifying replay is to compare the posterior probability of a specified trajectory segment given the spike trains intended for analysis with the marginal probability of the trajectory segment, and identify times at which the posterior probability obtains large maxima. Post hoc tests of significance are not required since variability in trajectories is captured by our model.

The methods presented here are well-suited to the study of replay even in problematic data conditions such as small neuronal sample size. With further scope for development, in particular in respect to the way we construct template trajectories for detection, we propose to use these methods to explore the open questions about the phenomenon of replay, such as the role of time compression, the details of replay episodes of varying temporal and spatial characteristics and how these relate to the experiences or cognitive demands of the animal, and the coordination of replay events between different parts of the brain.

## Appendix A: Convex space position transformation and distance metric $\bar {d}$

To compute the distance $\bar {d}(x^{\prime }, x^{\prime \prime })$ for each $x^{\prime }, x^{\prime \prime } \in \{ 1, 2, \dots , M \}$, we consider the discretised environment as a graph with discrete positions constituting the nodes and an edge connects every pair of nodes for which the corresponding positions are adjacent horizontally, vertically or diagonally. Edges are weighted by the distance between the centroids of the corresponding positions. Then $\bar {d} \left (x^{\prime }, x^{\prime \prime } \right )$ is the sum of the weights of the edges that form the shortest path from $x^{\prime }$ to $x^{\prime \prime }$. The shortest paths between every pair of nodes on the graph can be computed efficiently using the Floyd-Warshall algorithm or Johnson’s algorithm (Leiserson et al. 2001).

Our transformation $\mathbf {f}_{x} : \{1, 2, \dots , M\} \to \mathbb {R}^{2}$, with *locus* $x \in \{1, 2, \dots , M\}$, effects a change of basis for spatial coordinates relative to *x*. It is defined by

36 $$ \mathbf{f}_{x} \left(x^{\prime} \right) :\!= \frac{\bar{d} \left(x, x^{\prime} \right)}{d \left(x, x^{\prime} \right)} \left(x^{\prime} - x \right), $$

where $d \left (x, x^{\prime } \right )$ is the Euclidean distance between *x* and $x^{\prime }$.

## Appendix B: Posterior parameter sampling distributions

### B.1: Spike train model parameters

We consider the posterior distribution at time step *t* for parameter λ_*i*, *n*_. We have

$$\begin{array}{@{}rcl@{}} &&p \left(\lambda_{i, n} \mid x_{1:t}, \mathbf{y}_{1:t}, s_{0:t}, \mathbf{\theta}, \kappa, \mathbf{\phi} \right) \\ &&\qquad \propto p \left(\mathbf{y}_{1:t} \mid s_{0:t}, \mathbf{\theta} \right) p \left(\lambda_{i, n} \mid \mathbf{\phi}, \kappa \right) \\ &&\qquad \propto \left(\prod\limits_{u \le t: s_{u} = i}\! \frac{ \exp \left\{ -\delta t \lambda_{i, n}\right\}}{ y_{u, n}! } \left(\delta t \lambda_{i, n} \right)^{y_{u, n}} \!\! \right) \lambda_{i, n}^{\alpha - 1} \exp \left\{ -\lambda_{i, n} \beta\right\} \\ &&\qquad \propto \left(\prod\limits_{u \le t: s_{u} = i} \exp \left\{ -\delta t \lambda_{i, n} \right\} \lambda_{i, n}^{y_{u, n}} \right) \lambda_{i, n}^{\alpha - 1} \exp \left\{ -\lambda_{i, n} \beta\right\} \\ &&\qquad = \exp \left\{ -\left(\delta t c_{i, t} + \beta \right) \lambda_{i, n} \right\} \lambda_{i, n}^{\sum\limits_{u \le t: s_{u} = i} y_{u, n} + \alpha - 1}, \end{array} $$

which is, up to a normalising constant, the pdf of $\texttt {Gam}\left (\lambda _{i, n}; \alpha ^{*}, \beta ^{*} \right )$ with shape, rate parameterisation.

### B.2: Position model parameters

We first state some properties of the transformation **f**_*x*_. We have

37 $$\begin{array}{@{}rcl@{}} \mathbf{f}_{x^{\prime}} \left(x^{\prime\prime} \right) &= & - \mathbf{f}_{x^{\prime\prime}} \left(x^{\prime} \right) \qquad \text{and} \end{array} $$

38 $$\begin{array}{@{}rcl@{}} \mathbf{f}_{x^{\prime}} \left(x^{\prime\prime} \right) &= & \mathbf{f}_{x^{\prime}} \left(x^{\prime\prime\prime} \right) + \mathbf{f}_{x^{\prime\prime\prime}} \left(x^{\prime\prime} \right). \end{array} $$

Both are properties of vectors in $\mathbb {R}^{2}$. From Eqs. (11) and (12) we have

39 $$\begin{array}{@{}rcl@{}} && p \left(\xi_{i} \mid x_{1:t}, \mathbf{y}_{1:t}, s_{0:t}, \mathbf{\theta}, \kappa, \mathbf{\phi} \right) \\ & & \propto \exp \left\{\sum\limits_{u \le t: s_{u} = i} \mathbf{f}_{\xi_{i}} \left(x_{u} \right)^{\intercal} \Sigma_{i}^{-1} \mathbf{f}_{\xi_{i}} \left(x_{u} \right)\right\} \\ && = \exp \left\{\sum\limits_{u \le t: s_{u} = i} \left(\mathbf{f}_{\xi^{*}} \left(x_{u} \right) - \mathbf{f}_{\xi^{*}} \left(\xi_{i} \right) \right)^{\intercal} \Sigma_{i}^{-1} \left(\mathbf{f}_{\xi^{*}} \left(x_{u} \right) - \mathbf{f}_{\xi^{*}} \left(\xi_{i} \right) \right)\right\} ,\\ \end{array} $$

in which $^{\intercal }$ denotes the transpose operator. The exponent expands as

40 $$\begin{array}{@{}rcl@{}} && c_{i, t} \mathbf{f}_{\xi^{*}} \left(\xi_{i} \right)^{\intercal} \Sigma_{i}^{-1} \mathbf{f}_{\xi^{*}} \left(\xi_{i} \right) \\ &&\qquad - 2c_{i, t} \mathbf{f}_{\xi^{*}} \left(\xi_{i} \right) \Sigma_{i}^{-1} c_{i, t}^{-1} \sum\limits_{u \le t: s_{u} = i} \mathbf{f}_{\xi^{*}} \left(x_{u} \right) \\ &&\qquad + \sum\limits_{u \le t: s_{u} = i} \mathbf{f}_{\xi^{*}} \left(x_{u} \right)^{\intercal} \Sigma_{i}^{-1} \mathbf{f}_{\xi^{*}} \left(x_{u} \right). \end{array} $$

Now we obtain the form of the Gaussian posterior by completing the square. The exponent becomes

41 $$\begin{array}{@{}rcl@{}} & & c_{i, t} \left(c_{i, t}^{-1} \sum\limits_{u \le t: s_{u} = i} \mathbf{f}_{\xi^{*}} \left(x_{u} \right) - \mathbf{f}_{\xi^{*}} \left(\xi_{i} \right) \right)^{\intercal} \\ &&\qquad \times \Sigma_{i}^{-1} \left(c_{i, t}^{-1} \sum\limits_{u \le t: s_{u} = i} \mathbf{f}_{\xi^{*}} \left(x_{u} \right) - \mathbf{f}_{\xi^{*}} \left(\xi_{i} \right) \right) \\ &&\qquad + \sum\limits_{u \le t: s_{u} = i} \mathbf{f}_{\xi^{*}} \left(x_{u} \right)^{\intercal} \Sigma_{i}^{-1} \mathbf{f}_{\xi^{*}} \left(x_{u} \right) \\ &&\qquad - c_{i, t} \left(c_{i, t}^{-1} \sum\limits_{u \le t: s_{u} = i} \mathbf{f}_{\xi^{*}} \left(x_{u} \right) \right)^{\intercal} \\ &&\qquad \times \Sigma_{i}^{-1} \left(c_{i, t}^{-1} \sum\limits_{u \le t: s_{u} = i} \mathbf{f}_{\xi^{*}} \left(x_{u} \right) \right), \end{array} $$

but the last two terms do not depend on *ξ*_*i*_ and so the posterior is, up to a normalising constant,

42 $$\begin{array}{@{}rcl@{}} & \exp \left\{ c_{i, t} \left(c_{i, t}^{-1} \sum\limits_{u \le t: s_{u} = i} \mathbf{f}_{\xi^{*}} \left(x_{u} \right) - \mathbf{f}_{\xi^{*}} \left(\xi_{i} \right) \right)^{\intercal} \right.\\ &\qquad \left. {} \times \Sigma_{i}^{-1} \left(c_{i, t}^{-1} \sum\limits_{u \le t: s_{u} = i} \mathbf{f}_{\xi^{*}} \left(x_{u} \right) - \mathbf{f}_{\xi^{*}} \left(\xi_{i} \right) \right) \right\} \\ & = \exp \left\{ c_{i, t} \left(\mathbf{f}_{\xi^{*}} \left(\xi_{i} \right) \right)^{\intercal} \Sigma_{i}^{-1} \left(\mathbf{f}_{\xi^{*}} \left(\xi_{i} \right) \right)\right\} , \end{array} $$

if we choose $\xi ^{*} = \bar {x}_{i}$, where $\bar {x}_{i}$ satisfies $c_{i, t}^{-1} {\sum }_{u \le t: s_{u} = i} \mathbf {f}_{\bar {x}_{i}} \left (x_{u} \right ) = 0$. In practise there may not be a solution due to the discretisation of space, so we take a value for $\bar {x}_{i}$ that minimises this expression as per Eq. (13).

### B.3: Rows of the transition matrix

We use the algorithm of Wong (1998) to sample **P**_*i*,⋅_ from the Generalised Dirichlet distribution with parameter vectors **ζ**_*i*_, **γ**_*i*_. The Generalised Dirichlet distribution can be constructed as a product of Beta distributions with parameters *ζ*_*i*, *j*_, *η*_*i*, *j*_ for 1 ≤ *j* ≤ *κ*, from which the **γ**_*i*_ parameters can be derived as:

43 $$\begin{array}{@{}rcl@{}} \gamma_{i, \kappa} & = & \eta_{i, \kappa} - 1, \\ \gamma_{i, j} &= & \eta_{i, j} - \zeta_{i, j + 1} - \eta_{i, j + 1} \qquad \! \text{for } j = \kappa - 1, \dots, 1 \end{array} $$

(for details see Wong 1998). Therefore, if we set

44 $$\begin{array}{@{}rcl@{}} \eta_{i, \kappa} &= & \gamma_{i, \kappa}(t) + 1, \\ \eta_{i, j} &= & \gamma_{i, j}(t) + \zeta_{i, j + 1} + \eta_{i, j + 1} \qquad \text{for } j = \kappa - 1, \dots, 1, \end{array} $$

we retrieve the parameters of the underlying Beta distributions, and we can use the following procedure to sample **P**_*i*,⋅_:

- sample $P_{i, 1} \sim \texttt {Beta} \left (\zeta _{i, 1}, \eta _{i, 1} \right )$
- set *σ*←*P*_*i*,1_
- for *j* from 2 to *κ*:
  - sample $P_{i, j} \sim \texttt {Beta} \left (\zeta _{i, j}, \eta _{i, j} \right )$
  - then $P_{i, j} \gets P_{i, j} \left (1 - \sigma \right )$
  - set *σ*←*σ*+*P*_*i*, *j*_

## Appendix C: Sequential Monte Carlo (SMC) algorithm for Bayesian parameter inference

This section describes the SMC algorithm of Chopin (2007) to sample from the sequence of posterior distributions $p \left (\mathbf {\theta }, \kappa \mid x_{1:t}, \mathbf {y}_{1:t}, \mathbf {\phi } \right )$, where 1 ≤ *t* ≤ *T*.

- *Initialisation:* Use Eq. (7) to sample *κ*, and **𝜃** conditional on sampled values of *κ*, *H* times, obtaining $\{\mathbf {\theta }^{h}, \kappa ^{h}\}_{h = 1}^{H}$. We refer to the set of all particles that sample the same value of *κ* as the *subpopulation corresponding to* *κ*. Initialise the *particle weights* as
  45 $$ w_{h} \gets \frac{1}{H} \qquad \text{for} \qquad h = 1, 2, \dots, H. $$
- *Loop:* At each time step *t* from 1 to *T*, perform all or some of the following tasks as necessary:

1. *Update weights:* Set
  46 $$ w_{h} \gets w_{h} p \left(x_{t}, \mathbf{y}_{t} \mid x_{1:t - 1}, \mathbf{y}_{1:t - 1}, \mathbf{\theta}^{h} \kappa^{h} \right) $$
  for $h = 1, 2, \dots , H$. The weight update factor is the ratio of *data* likelihoods at subsequent time steps:
  47 $$ p \left(x_{t}, \mathbf{y}_{t} \mid x_{1:t - 1}, \mathbf{y}_{1:t - 1}, \mathbf{\theta}^{h}, \kappa^{h} \right) = \frac{ p \left(x_{1:t}, \mathbf{y}_{1:t} \mid \mathbf{\theta}^{h}, \kappa^{h} \right) }{ p \left(x_{1:t - 1}, \mathbf{y}_{1:t - 1} \mid \mathbf{\theta}^{h}, \kappa^{h} \right) } $$
  (using $p \left (x_{1}, \mathbf {y}_{1} \mid \mathbf {\theta }^{h}, \kappa ^{h} \right )$ at *t* = 1). The data likelihood at *t* can be computed by marginalising $\widetilde {S}_{t}$ from the *forward function at t*, $p \left (\widetilde {S}_{t}, x_{1:t}, \mathbf {y}_{1:t} \mid \mathbf {\theta }^{h}, \kappa ^{h} \right )$, computed using the *forward recursions*, explained in Scott (2002).
2. *Check for sample degeneracy:* evaluate the ESS (33) using the sample variance of the weights. If *ESS* exceeds a threshold *ESS* ^∗^, skip *(3)* and *(4)* and proceed to the next time step. Chopin (2007) suggests a threshold of $ESS^{*} = \frac {H}{2}$.
3. *Resample with positive discrimination and reset weights:* resample particles according to their weights (using, for example, the *residual resampling* approach of Liu and Chen 1998). This should be done in conjunction with the “positive discrimination” scheme of Chopin (2007), to make it more likely we retain some particles in each subpopulation after resampling. Compute the sample approximation to the marginal (partial) posterior over *κ*:
  48 $$ \hat{p}_{\kappa^{\prime}, t} :\!= p \left(\kappa = \kappa^{\prime} \mid x_{1:t}, \mathbf{y}_{1:t}, \mathbf{\phi} \right) = \frac{ {\sum}_{h: \kappa^{h} = \kappa^{\prime}} w_{h} }{{\sum}_{h = 1}^{H} w_{h} } $$
  for each $1 \le \kappa ^{\prime } \le \bar {\kappa }$. If $\hat {p}_{\kappa ^{\prime }, t}H$ falls below a tolerance level *H* ^∗^, resample *H* ^∗^ times from the subpopulation corresponding $\kappa ^{\prime }$. For these resampled particles, set
  49 $$ w_{h} \gets \frac{ \hat{p}_{\kappa^{\prime}, t} H }{ H^{*} } < 1. $$
  We thus give discriminated particles lower importance, compensating for the biasing effect of their preferential retention. Chopin (2007) suggests to use $H^{*} = \frac {H}{10}$.
  After resampling within all subpopulations requiring positive discrimination, resample the remaining particles maintaining a sample size of *H* and set their weights to 1, then normalise all weights.
4. *“Move” particles using a single sweep of Gibbs sampling:* for each 1 ≤ *h* ≤ *H*, sample $\widetilde {s}_{0:t}^{h}$ according to the distribution $p \left (\widetilde {s}_{0:t} \mid x_{1:t}, \mathbf {y}_{1:t}, \mathbf {\theta }^{h}, \kappa ^{h} \right )$ using the *stochastic backward recursions* (described in Scott 2002), then sample *𝜃* ^*h*^ according to the posterior $p \left (\mathbf {\theta }_{t} \mid s_{0:t}^{h}, x_{1:t}, \mathbf {y}_{1:t}, \mathbf {\theta }^{h}, \kappa ^{h} \right )$ described in Section 2.3.

By our use of conjugate priors, we are only required to compute the statistics $\mathbf {A}(t), \mathbf {B}(t), c_{i, t}, SS\left (i, t, \xi _{i} \right )$ and $\bar {x}_{i}$ for 1 ≤ *i* ≤ *κ*, then to sample from standard distributions. We perform sampling from the Gamma, Inverse-Wishart, and Beta distributions using built-in functions of software package MATLAB.

## Appendix D: Viterbi-like algorithm for decoding position

Here is described a recursive algorithm to find

50 $$ \hat{x}_{1:T} = \underset{x_{1:T}}{\arg\max} p \left(x_{1:T}, \mathbf{y}_{1:T}, \theta \right) = \arg\max\limits_{x_{1:T}} p \left(x_{1:T} \mid \mathbf{y}_{1:T}, \theta \right). $$

First, define

51 $$ V_{t} \left(v, j \right) :\!= \max\limits_{x_{1:t - 1}} \left\{ p \left(S_{t} = j, X_{1:t - 1} = x_{1:t - 1}, X_{t} = v, \mathbf{y}_{1:t}, \theta \right) \right\} . $$

Now notice that

52 $$\begin{array}{@{}rcl@{}} V_{t} \left(v, j \right) &= &\max\limits_{x_{1:t - 1}} \left\{ \sum\limits_{i = 1}^{\kappa} p \left(S_{t} = j \mid S_{t - 1} = i, \theta \right) \right.\\ &&\left. \times p \left(S_{t - 1} = i, X_{1:t - 2} = x_{1:t - 2}, X_{t - 1} = x_{t - 1}, \mathbf{y}_{1:t - 1} \mid \theta \right) \vphantom{\sum\limits_{i = 1}^{\kappa}} \right\}\\ &&\times p \left(\mathbf{y}_{t} \mid S_{t} = j, \theta \right) p \left(X_{t} = v \mid S_{t} = j, \theta \right), \end{array} $$

by the conditional independence structure and since the last two terms do not depend on *x*_1:*t*−1_. This suggests the recursions

53 $$\begin{array}{@{}rcl@{}} V_{t} \left(v, j \right) &= &\max\limits_{u} \left\{ \sum\limits_{i = 1}^{\kappa} p \left(S_{t} \,=\, j \mid S_{t - 1} = i, \theta \right) V_{t - 1} \left(u, i \right) \right\}\\ &&\times p \left(\mathbf{y}_{t} \mid S_{t} = j, \theta \right) p \left(X_{t} = v \mid S_{t} = j, \theta \right),\\ \end{array} $$

for *t* from 2 to *T*, with initialisation

54 $$\begin{array}{@{}rcl@{}} V_{1} \left(v, j \right) &= &\sum\limits_{i = 1}^{\kappa} p \left(S_{1} = j \mid S_{0} = i \right) p \left(S_{0} = i \mid \theta \right) \\ &&\times p \left(\mathbf{y}_{1} \mid S_{1} \,=\, j, \theta \right) p \left(X_{1} \,=\, v \mid S_{1} \,=\, j, \theta \right). \end{array} $$

Once these have been computed for each $v \in \left \{ 1, 2, \dots , M \right \} $ and each $j \in \left \{1, 2, \dots , \kappa \right \} $ we can use the recursions

55 $$\begin{array}{@{}rcl@{}} \hat{x}_{t} = \arg\max\limits_{v} &\sum\limits_{j = 1}^{\kappa} p \left(X_{t + 1} = \hat{x}_{t + 1} \mid S_{t + 1} = j, \theta \right) \\ &\quad\times \sum\limits_{i = 1}^{\kappa} p \left(S_{t + 1} \!= j \mid S_{t} = i, \theta \right) V_{t} \left(v, i \right) \end{array} $$

for *t* from *T*−1 to 1, with initialisation

56 $$ \hat{x}_{T} = \arg\max\limits_{v} \sum\limits_{j = 1}^{\kappa} V_{T} \left(v, j \right). $$

## Appendix E: Algorithm for computing the posterior probability of a trajectory

Here is described an efficient recursive algorithm for computing the posterior probability of a position trajectory *x*_1:*a*_ of length *a* at any offset *t*; that is

57 $$ p \left(X_{t} = x_{1}, X_{t + 1} = x_{2}, \dots, X_{t + a - 1} = x_{a} \mid \mathbf{y}_{1:T}, \theta \right) $$

for any $t \in \left \{ 1, 2, \dots , T - a + 1\right \} $.

We begin by performing the forward and backward recursions; then the algorithm has two stages. First we compute the intermediary quantities

58 $$ p \left(S_{t + a - 1} = i, X_{t} = x_{1}, X_{t + 1} = x_{2}, \dots, X_{t + a - 1} = x_{a}, \mathbf{y}_{1:t + a - 1} \mid \theta \right) $$

for each $i \in \left \{1, 2, \dots , \kappa \right \} $ using the forward accumulation steps

59 $$\begin{array}{@{}rcl@{}} &&p \left(S_{t + u - 1} = j, X_{t} = x_{1}, \dots, X_{t + u - 1} = x_{u}, \mathbf{y}_{1:t + u - 1} \mid \theta \right) \\ &&\qquad = p \left(X = x_{u} \mid S = j, \theta \right) p \left(\mathbf{y}_{t + u - 1} \mid S_{t + u - 1} = j, \theta \right) \\ &&\qquad \quad\times \sum\limits_{i = 1}^{\kappa} p \left(S_{t + u - 1} = j \mid S_{t + u - 2} = i, \theta \right) \\ &&\qquad \quad\times p \left(S_{t + u - 2} \,=\, i, X_{t} \,=\, x_{1}, \dots, X_{t + u - 2} \,=\, x_{u - 1}, \mathbf{y}_{1:t + u - 2} \mid \theta \right)\\ \end{array} $$

for *u* from 2 to *a* with initialisation

60 $$\begin{array}{@{}rcl@{}} &&p \left(S_{t} = j, X_{t} = x_{1}, \mathbf{y}_{1:t} \mid \theta \right) \\ &&\qquad = p \left(X = x_{1} \mid S = j, \theta \right) p \left(S_{t} = j, \mathbf{y}_{1:t} \mid \theta \right), \end{array} $$

where $p \left (S_{t} = j, \mathbf {y}_{1:t} \mid \theta \right )$ is the *t*th forward function evaluated at state *j*. Then we perform the second stage:

61 $$\begin{array}{@{}rcl@{}} &&p \left(X_{t} = x_{1}, X_{t + 1} = x_{2}, \dots, X_{t + a - 1} = x_{a} \mid \mathbf{y}_{1:T}, \theta \right) \\ &&\propto \sum\limits_{j = 1}^{\kappa} p (S_{t + a - 1} = i, X_{t} = x_{1}, X_{t + 1} = x_{2}, \dots,\\ &&\qquad\quad X_{t + a - 1} = x_{a}, \mathbf{y}_{1:t + a - 1} \mid \theta ) \\ &&\qquad \times p \left(\mathbf{y}_{t + a:T} \mid S_{t + a - 1} = j, \theta \right), \end{array} $$

in which $p \left (\mathbf {y}_{t + a:T} \mid S_{t + a - 1} = j, \theta \right )$ is the $\left (T - t - a + 1 \right )$th backward function evaluated at state *j*, and with normalising constant

62 $$ p \left(\mathbf{y}_{1:T} \mid \theta \right) = \sum\limits_{j = 1}^{\kappa} p \left(S_{T} = j, \mathbf{y}_{1:T} \mid \theta \right). $$

For computing the above over all possible *t*, the time and memory requirements are proportional to those of the forward-backward algorithm.

## Appendix F: Algorithm for computing the marginal probability of a trajectory

Here is described a recursive algorithm for computing the marginal probability of a position trajectory *x*_1:*a*_ of length *a* at any offset *t*,

63 $$ p \left(X_{t} = x_{1}, X_{t + 1} = x_{2}, \dots, X_{t + a - 1} = x_{a} \mid \theta \right) $$

for any $t \in \left \{ 1, 2, \dots , T - a + 1 \right \} $. Using the model conditional distribution over positions given state and the state transition matrix, we recursively compute the quantities

64 $$\begin{array}{@{}rcl@{}} &&p \left(S_{t + u - 1} \!= j, X_{t} \,=\, x_{1}, X_{t + 1} \,=\, x_{2}, \dots, X_{t + u - 1} \,=\, x_{u} \mid \theta \right) \\ &&\quad = \sum\limits_{i = 1}^ \kappa P_{i, j} p \left(X_{t + u - 1} = x_{u} \mid S_{t + u - 1} = j, \theta \right) \\ &&\qquad \times p (S_{t + u - 2} = i, X_{t} = x_{1}, X_{t + 1} = x_{2}, \dots,\\ &&\qquad\qquad X_{t + u - 2} = x_{u - 1} \mid \theta ) \end{array} $$

for each $j \in \left \{1, 2, \dots , \kappa \right \} $ and for *u* from 2 to *a*. We assume (as discussed in Section 2.4.3) that by *t* the Markov chain has reached its equilibrium distribution *ν* so that we can initialise the algorithm with

65 $$ p \left(S_{t} = j, X_{t} = x_{1} \mid \theta \right) = \nu_{j} p \left(X_{t} = x_{1} \mid S_{t} = j, \theta \right) $$

for each $j \in \left \{ 1, 2, \dots , \kappa \right \} $. After performing the above recursions, we obtain the desired quantity with:

66 $$\begin{array}{@{}rcl@{}} &&p \left(X_{t} = x_{1}, X_{t + 1} = x_{2}, \dots, X_{t + a - 1} = x_{a} \mid \theta \right) \\ &&\qquad = \sum\limits_{i = 1}^ \kappa p (S_{t + a - 1} = i, X_{t} = x_{1}, X_{t + 1} = x_{2}, \dots,\\ &&\qquad\qquad\quad\,\,\, X_{t + a - 1} = x_{a} \mid \theta ). \end{array} $$

## Appendix G: Implementation of models BD and LP

Model BD assumes conditional independence of spike trains given position, and assumes positions are iid. The spike count in each time bin is distributed as Poisson, conditionally on position, and each position is a categorical random variable. The complete model likelihood factorises as

67 $$ p \left(\mathbf{y}_{1:T}, x_{1:T} \mid \lambda^{X}, \pi^{X} \right) \,=\, \prod\limits_{t = 1}^{T} p \left(\mathbf{y}_{t} \mid x_{t}, \lambda^{X} \right) \! p \left(x_{t} \mid \pi^{X} \right), $$

in which λ^*X*^ is a matrix of mean spike rates for each neuron and each position, and *π* ^*X*^ is the vector of categorical position outcome probabilities. Decoding is perfomed using the posterior distribution over position given spikes, using fitted values of parameters, i.e.

68 $$\begin{array}{@{}rcl@{}} &&p \left(X_{t} = x \mid \mathbf{y}_{t}, \hat{\lambda}^{X}, \hat{\pi}^{X} \right) \propto p \left(\mathbf{y}_{t} \mid X_{t} = x, \hat{\lambda}^{X}, \hat{\pi}^{X} \right) \\ &&p \left(X_{t} = x \mid \hat{\lambda}^{X}, \hat{\pi}^{X} \right). \end{array} $$

Parameters are inferred using maximum likelihood. MLE estimates using training data *x*_1:*T*_, **y**_1:*T*_ are

69 $$\begin{array}{@{}rcl@{}} &&\hat{\lambda}_{x, n} = \frac{{\sum}_{t: x_{t} = x} y_{t, n}}{\delta t {\sum}_{t = 1}^{T} \bullet \left\{x_{t} = x \right\}}, \end{array} $$

70 $$\begin{array}{@{}rcl@{}} &&\hat{\pi}^{X}_{x} = \frac{{C_{T}^{X}} \left(x \right)} {T}, \end{array} $$

71 $$\begin{array}{@{}rcl@{}} &&\text{for } n = 1, 2, \dots, C, \quad x = 1, 2, \dots, M. \end{array} $$

(see, e.g. Zhang et al. (1998), in which the former is named the “spatial occupancy map”). We note that Zhang et al. (1998) performed smoothing of firing rates using a Gaussian kernel, which can have a significant impact on decoding performance when little data is available. In our experiments this smoothing was found not to make a significant different to decoding performance, and so we compared against the BD method with no smoothing as a default setting.

LP differs from BD in assuming position variables *X*_1:*T*_ have the Markov property, rather than being iid; that is, *X*_*i*_⊥*X*_*t*:*i*−2_∣*X*_*t*−1_ for $t = 2, 3, \dots , T$. The complete model likelihood is thus

72 $$\begin{array}{@{}rcl@{}} p \left(\mathbf{y}_{1:T}, x_{1:T} \mid \lambda^{X}, \pi^{X}, Q \right) &=& p \left(\mathbf{y}_{1} \mid x_{1}, \lambda^{X} \right) \! p \left(x_{1} \!\mid\! \pi^{X} \right)\\ && \times \prod\limits_{t = 2}^{T} p \left(\mathbf{y}_{t} \mid x_{t}, \lambda^{X} \right) \\ &&\qquad\, p \left(x_{t} \mid x_{t - 1}, Q \right), \end{array} $$

with parameters λ^*X*^, *π* ^*X*^ the same as in BD and transition matrix *Q*. Rows of *Q* - the probabilities of transitioning to each position in the maze (*destination*) conditional on the position at the previous time step (*source*) - are normalised densities of a 1-D Gaussian centered on the source with zero mean and standard deviation ${\sigma ^{X}_{x}}$ for each source *x*. That is, Euclidean distance $d\left (X_{t}, X_{t - 1} = x \right )$, conditional on *X*_*t*−1_ = *x*, is distributed as

73 $$\begin{array}{@{}rcl@{}} d \left(X_{t}, X_{t - 1} \,=\, x \right) \!\sim\! N\! \left(0; {\sigma^{X}_{x}} \right), \,\,\,\, i \,=\, 1, 2, \dots, T,\,\,\,\, x \,=\, 1, 2, \dots, M.\\ \end{array} $$

Then, elements of each row *Q*_*x*,⋅_ of the transition matrix are assigned probability densities from this Gaussian and normalised so that the row sum is 1. The MLE estimates are given by

74 $$ \hat{\sigma}^{X}_{x} = \sqrt{\frac{{\sum}_{t > 1: x_{t - 1} = x} d \left(x_{t - 1}, x_{t} \right)^ 2}{{\sum}_{t = 2}^{T} \bullet \left\{x_{t} = x \right\}}}, \quad x = 1, 2, \dots, M. $$

MAP decoding is achieved in LP using the forward (for filtered estimates) or forward-backward algorithm (smoothed estimates) (see Scott 2002); the most probable sequence of positions given spike trains (Viterbi path) can be obtained using the standard Viterbi algorithm (Viterbi 1967).
